# Characterization of Light Lesion Paradigms and Optical Coherence Tomography as Tools to Study Adult Retina Regeneration in Zebrafish

**DOI:** 10.1371/journal.pone.0080483

**Published:** 2013-11-26

**Authors:** Anke Weber, Sarah Hochmann, Peter Cimalla, Maria Gärtner, Veronika Kuscha, Stefan Hans, Michaela Geffarth, Jan Kaslin, Edmund Koch, Michael Brand

**Affiliations:** 1 DFG-Research Center for Regenerative Therapies Dresden/Cluster of Excellence (CRTD), Biotechnology Center, Technische Universität Dresden, Dresden, Germany; 2 Medical Faculty Carl Gustav Carus, Technische Universität Dresden, Dresden, Germany; University Zürich, Switzerland

## Abstract

Light-induced lesions are a powerful tool to study the amazing ability of photoreceptors to regenerate in the adult zebrafish retina. However, the specificity of the lesion towards photoreceptors or regional differences within the retina are still incompletely understood. We therefore characterized the process of degeneration and regeneration in an established paradigm, using intense white light from a fluorescence lamp on swimming fish (diffuse light lesion). We also designed a new light lesion paradigm where light is focused through a microscope onto the retina of an immobilized fish (focused light lesion). Focused light lesion has the advantage of creating a locally restricted area of damage, with the additional benefit of an untreated control eye in the same animal. In both paradigms, cell death is observed as an immediate early response, and proliferation is initiated around 2 days post lesion (dpl), peaking at 3 dpl. We furthermore find that two photoreceptor subtypes (UV and blue sensitive cones) are more susceptible towards intense white light than red/green double cones and rods. We also observed specific differences within light lesioned areas with respect to the process of photoreceptor degeneration: UV cone debris is removed later than any other type of photoreceptor in light lesions. Unspecific damage to retinal neurons occurs at the center of a focused light lesion territory, but not in the diffuse light lesion areas. We simulated the fish eye optical properties using software simulation, and show that the optical properties may explain the light lesion patterns that we observe. Furthermore, as a new tool to study retinal degeneration and regeneration in individual fish *in vivo*, we use spectral domain optical coherence tomography. Collectively, the light lesion and imaging assays described here represent powerful tools for studying degeneration and regeneration processes in the adult zebrafish retina.

## Introduction

The teleost retina is closely related to the human retina, both in respect to its cell types and their laminated arrangement. In contrast to mammals, however, zebrafish can efficiently regenerate injuries to the retina, a better understanding of which might open up new regenerative therapies for retinal diseases (reviewed by [1]). Following injury of the adult retina, zebrafish can regenerate [2], [3] by activating Müller glia (MG) to produce progenitor cells replacing all cell types of the neuronal retina, including photoreceptor cells and mature neurons [4]–[6].

The regeneration of fish retina has been studied using different lesion paradigms such as: genetic [7], surgical [8], cytotoxic [5] stab [6], nitroreductase mediated[9] and light induced cell death paradigms [4], [10]. To elucidate the molecular and cellular pathways involved in regeneration, it is particularly convenient to induce photoreceptor-selective damage by non-invasive methods, like intense light application. The mechanisms of phototoxicity, photoreceptor degeneration and DNA repair response have been studied in rats [11], [12]. Continuous exposure to bright white light leads to rhodopsin bleaching and oxidative stress that result in cell death of photoreceptors (reviewed by [13]). In zebrafish, two different paradigms have previously been used to induce damage to photoreceptors in the adult zebrafish retina. One paradigm used long exposures to bright halogen light (∼20 000 lux total light intensity) on dark-adapted albino animals, and comprehensive studies of lesion characteristics with respect to regional differences, affected cell types and cell death time course have been performed [10], [14]. Characteristic for these light lesions is the extensive cell death of photoreceptors in the central and dorsal, but not in the ventral part of the retina. Furthermore, a correlation of photoreceptor damage to induced proliferation in the INL and upregulation of α1-tubulin in MG was found. A significant drawback of this lesion paradigm is the long exposure time of up to 7 continuous days. The second paradigm used short exposure for only 30′ to strong UV light from a mercury arc lamp (∼180 000 lux) to treat normally pigmented fish [4], [15]. This treatment exposed fish to ∼10-fold higher light intensity compared to the halogen lamps used for continuous exposure. Although this paradigm has been used in several studies [10], [16], [17], only recently more details on lesion characteristics were described [18]. The data obtained from non-pigmented albino fish show that more rods than cones were lost in a predefined, small area of the retina. Damage to the ventral area of the retina was either relatively mild or not at all observed throughout all studies on light lesions.

A common feature for both assays is the loss of photoreceptors after light exposure as well as a robust regeneration response [4], [10], [14], [19]. However it remains unclear whether all photoreceptor subtypes are equally damaged throughout the retina and to which extent other cell types are affected by light in normally pigmented fish.

In this study, we have characterized in detail the already published paradigm using short exposure for only 30′ to strong UV light from a mercury arc lamp [4], [15], [18] – which will be referred to as diffuse light lesion paradigm – and established a new light lesion paradigm that allows us to generate small lesions in the retina. We compare both assays by characterizing the morphological details of regional damage response, cell death and proliferation as well as the effects on other cell types adjacent to photoreceptors. The new focused light lesion paradigm that we developed allows us to study precisely reproducible lesions that will be useful for future analysis of molecular pathways in retinal degeneration and regeneration.

## Methods

### Ethics statement

All animal experiments were carried out in strict accordance with European Union and German laws (Tierschutzgesetz). All experimental procedures were approved by the animal ethics committee of the TU Dresden and the Landesdirektion Sachsen (approval number: AZ 24D-9168.11-1/2008-4 and 24-9168.11-1/2013-5). This institutional review board specifically approved this study.

### Fish Maintenance

Fish were kept under standard conditions as previously described [20]. Wild-type (WT) experimental animals were adult fish from the gol-b1 line in the AB genetic background [21]. Adult fish were 6–8 months old and had a 24–32 mm body length. We used the following transgenic reporter lines expressing GFP under a tissue specific promoter: Tg(−3.7rho:EGFP)kj2 [22] abbreviated as rh1:GFP, Tg(−5.5opn1sw1:EGFP)kj9 [23] (abb.: opn1sw1:GFP) and Tg(gfap:GFP)^∧^mi2001 [15] (abb.: gfap:GFP) in AB genetic background.

### Experimental procedure

**Figure 1:**
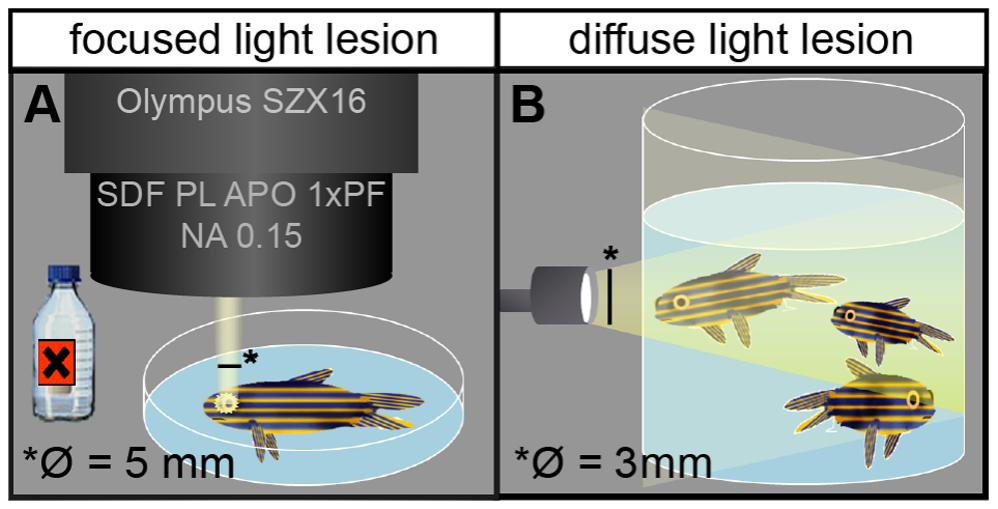
Schematic illustration of lesion paradigms. **A**: In focused light lesion the fish is anaesthetized under the stereoscope and exposed to light onto one eye from one single angle, producing a well circumscribed lesioned area in the illuminated retina, next to non-illuminated control areas. The non-illuminated eye serves as an additional internal control. **B**: In diffuse light lesions, fish swim freely in a beaker and are exposed to light from all angles and to both eyes.

All light lesion experiments were conducted with the EXFO X-Cite 120W metal halide lamp (EXFO Photonic Solutions, Mississauga, Ontario, Canada). We introduced a new light lesion method using a microscope to illuminate immobilized fish and named it focused light lesion in the subsequent text. For focused light lesions fish were briefly anaesthetized in 0.024% Tricaine (Sigma) in system water until they became unresponsive to touch. Next they were transferred to a petridish containing a piece of Whatman paper and 0.012% Tricaine in system water. Fish were placed under a stereomicroscope (Olympus SZX16, objective: SDF PL APO 1xPF) and the left eye was exposed to bright white light for 30 minutes (∼80 000 lux, Fig. 1A). The right eye was not exposed to bright light and serves as internal control. In diffuse light lesion experiments, fish were transferred to a 250 ml beaker containing system water and placed 3 cm in front of the light source for 30′ (∼200 000 lux, Fig. 1B, similar to [4], [15]). Afterwards all fish were returned to the system for recovery under normal light conditions.

### BrdU Labeling

To label cells in S-phase of the cell cycle, zebrafish were immersed in 10 mm BrdU (Sigma) solution [24]. The BrdU was dissolved in E3 medium and adjusted to pH 7.5. For BrdU-pulse experiments, a maximum of 4 fish were simultaneously immersed in 500 ml solution for two hours in BrdU and sacrificed immediately afterwards.

### Tissue Preparation

Fish heads were fixed at 4°C overnight in 4% paraformaldehyde/0.1 M phosphate buffer (PB), pH 7.5. They were transferred for decalcification and cryoprotection to 20% sucrose/20% EDTA in 0.1 M PB, pH 7.5 and incubated overnight at 4°C. Heads were frozen in 7.5% gelatine/20% sucrose and cut into 12–16 µm sections using a HM 50600 cryostat (respective section thickness is indicated in the text and figure legends). For paraffin sections processing was done in a Paraffin-Infiltration-Processor (STP 420, Zeiss) according to the following program: ddH_2_0: 1×1′; 50% ethanol (EtOH) 1×5′; 70% EtOH 1×10′; 96% EtOH 1×25′; 96% EtOH 2×20′; 100% EtOH 2×20′; xylene 2×20′; paraffin 3×40′/60°C; paraffin 1×60′/60°C. The heads were embedded using Embedding Center EG1160 (Leica). Semi-thin sections (1 µm) were cut on an Ultracut microtome (Mikrom) and counterstained using hematoxilin/eosin (HE, Sigma).

### Immunohistochemistry

Immunohistochemistry (IHC) was performed as previously described [24], [25]. Briefly, primary and secondary antibodies were incubated in PBS with 0.3% Triton X-100 (PBSTx). Primary antibodies were incubated overnight at 4°C and secondary antibodies for 2 h at room temperature. The slides were washed in PBSTx and mounted in 70% glycerol/PBS. Information about primary antibodies is given in Table S1. The secondary antibodies were Alexa 488-, 555- and 635- conjugated (Invitrogen, Karlsruhe). To retrieve the antigenicity of BrdU, antigen retrieval was performed with 2 M HCl for 15′ at 37°C and subsequent washing steps in sodium tetraborate buffer and PBSTx. Antigen retrieval for HuC/D was performed as described before [25].

All IHC were done on at least three individuals and all nuclei were counterstained with 4′,6-diamidino-2-phenylindole (DAPI 1 µg/ml, Invitrogen). Terminal deoxynucleotidyl transferase-mediated biotinylated UTP nick end labeling (TUNEL) assays were carried out using the ApopTag Red In Situ Apoptosis Detection Kit (Chemicon), according to the manufacturer's instructions. In every experimental series, positive [digestion with DNase I (3000-3 U/ml) in 50 mm Tris-HCl (pH 7.5), 1 mg/ml BSA] and negative (omitting the TdT enzyme) control slides were included.

### In Situ Hybridization

The full length *gad1b* gene (2505 bp, coding sequence from 346–2109 bp) was obtained from a plasmid kindly provided by Catherina Becker. We subcloned a 745 bp fragment within the coding region (748–1493 bp) into the corresponding sites of pBluescript vector and confirmed insertion by sequencing. Fragments of opsin coding regions were cloned from genomic DNA into the corresponding sites of pBluescript vector and confirmed by sequencing. Primers for amplification of *rho, opn1sw1, opn1lw1 and opn1sw2* are listed in Table S2. The plasmid containing *opn1mw1* was obtained from P. Raymond (Genbank Accession Number: AF109369)[26]. For probe synthesis, plasmids were linearized with EcoRI and Digoxigenin labelled RNA probes were transcribed with T3 RNA polymerase. *In situ* hybridization and probe generation was essentially performed as previously described [27]. All *in situ* hybridizations were done on at least three individuals.

### Image acquisition

Images were taken with ZEISS Axio Imager.Z1 microscopes and a Leica TCS-SP5 confocal microscope using HC PL APO CS 20/0.7 NA, HCX PL APO 40/1.25 NA and HCX PL APO 63/1.2 NA objectives. To minimize cross talk between the channels in multicolored specimens, sequential image acquisition was performed. Images were processed using Fiji and Adobe Photoshop CS5. Figures were assembled using Adobe Illustrator CS5.

### Cell counting and statistical analysis

We counted the number of BrdU+ cells in the whole retina in every fifth section (14 µm) and normalized it to the length of each individual section. Also, the number of TUNEL+ and L-Plastin+ cells in each layer was counted in every third section (12 µm) and normalized to the length of each individual section. Then we calculated the average of all positive cells per mm retinal length in each experimental group. At least 3 fish were used for each experiment. Quantifications of inner retinal neurons and MG were done in a central area of maximum damage within 200 µm of retina length on 3 consecutive sections (14 µm) for focused light lesion, and every 5^th^ section (14 µm) after diffuse light lesion. Quantification of photoreceptor lesion size was measured in sections with Fiji Software. The extent of rod lesions was determined as decrease in rh1 *in situ* signal of at least 50%. To ensure reproducible analysis of regions along the anterior-posterior axis we determined the absolute number of sections comprising the complete retina when collecting three series of sections (e. g. 36 sections per retina and slide). Next, anterior and posterior sections were determined (e.g. dividing the number of sections by three: 36/3 = 12 and counting section 11, 12, 13 (anterior), 17, 18, 19 (central) and 23, 24, 25 (posterior)).

To distinguish between the dorsal and the ventral retina, we have set the centre point of each retinal section as half of the complete circumference, splitting the retina into a dorsal and ventral half. To determine the size of the dorsal and ventral lesion, respectively, we measured the extent of the lesion from the centre point in ventral and dorsal direction. Lesioned area was normalized as % of total retina length in each section (16 µm).

Quantification of UV cones in flat-mounted retina samples was obtained from tile images of the whole retina in 5 optical sections with 2.8 µm thickness each. All of the following image processing was done in Fiji software [28]. Five optical sections per sample were combined in maximum intensity z-projections before using the Rolling Ball Background Subtraction Plugin (radius = 30). The image was converted to 8 bit and Auto local threshold determined (Method  =  Bernsen, Radius = 50). Then we inverted the image and used the watershed command to separate adjacent cells. Finally, cells were counted with “Analyze Particles” command (Size (pixel): 50-Infinity, Circularity: 0.40–1.00). Retinal area was measured in pixel^2^ and converted to mm^2^ according to the metadata of the TIFF file. Images of regenerated samples were cropped to the regenerated area excluding the undamaged ventral area. To analyze significance, p values were determined with GraphPad Prism using one-way ANOVA, followed by Tukey posttest. Error bars represent SEM. ***p≤0.001; **p≤0.01; *p≤0.05. p>0.05 was not considered significant.

### Lens simulation

Morphology of the fish eye was analyzed by photos of freshly dissected eyes under a stereomicroscope with the aid of scale paper. Eyes of at least 3 siblings of 29 mm body length each were measured. The diameter of the iris, pupil, lens and eyeball in proximo-distal orientation were determined. Additionally, we measured fish eye anatomy also in vivo by optical coherence tomography (OCT) imaging of anaesthetized fish in the same position as was used in focused light lesions.

We simulated the fish eye in Zemax software (Radiant Zemax, LLC, USA) by a complex of lenses having similar dimensions and optical properties as the fish eye, and by using non-sequential ray tracing. Two curved planes at the back of the ‘eye’-model act as detector to measure the amount of light reaching the nerve fibre layer and the RPE, respectively. The eye itself was modeled as a two half-spheres with refractive index properties of water to accord to the shape of the fish eye. According to several findings in the literature regarding the optical properties of fish lenses [29], [30], the zebrafish lens was simulated using a spherical gradient index lens with a polynomial refractive index profile. Using data extracted from dispersion measurements of an african cichlid fish lens (Haplochromis burtoni) [31], the refractive index n(r,λ), depending on the radial position r measured from the center of the lens, was assumed according to equation 1 for each given wavelength λ. 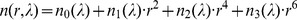
(1)


Lens dispersion was modeled with the reduced dispersion formula of the example model Gradient 6 from ZEMAX utilizing a nonlinear least square fit to obtain the coefficients A_i_, B_i_ and C_i_ for every parameter n_i_ in equation (1): 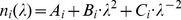
(2)


The parameters deduced for a lens of radius R = 0.48 mm are shown in table 1. With these parameters, the refractive power of the modeled fish lens immersed in water resulted in 820 dpt for a wavelength of 800 nm and an illumination aperture of 0.4 mm. This corresponds to a focal length of 1.22 mm measured from the center of the lens to the focal point. The effective refractive index, which was later used for the design of the custom made optics in the OCT setup, was determined to be 1.64.

**Table 1:** Parameters for lens simulation in Zemax software according to equation 2.

i	A	B/nm^−2^	C/nm^2^
0	1.545	−5.042•10^−8^	3317.542
1	−0.741	3.198•10^−7^	8948.224
2	−7.399•10^−12^	1.197•10^−17^	1.036•10^−6^
3	−2.047	−1.480•10^−6^	−58235.821

For simulating the impact of focused light onto the retina of the zebrafish, the illumination of a microscope was modeled by using a beam positioned at infinity with a Gaussian shaped intensity profile (beam width  = 2.5 mm; number of rays = 1 000 000).

The light beam from the microscope is not perfectly orthogonal, but tilted by 15.8° towards the dorsal side of the fish eye. The anatomy of the fish head also leads to a tilt of ∼18° towards the anterior side. These parameters were represented in our simulation by adjusting the angle of the light beam accordingly.

### Spectral Domain Optical Coherence Tomography

We use a self-developed spectral domain OCT system for simultaneous dual-band imaging in the 800 nm- and 1300 nm-wavelength range [32], the two most common spectral bands in biomedical OCT. The system is illuminated by a novel broadband supercontinuum laser source (SuperK Versa Super Continuum Source, Koheras A/S, Denmark), and enables the combination of high resolution short-wave imaging with long-wave enhanced penetration depth into the choroid and sclera at the posterior eye segment. The axial resolutions in tissue were measured to be 3.2 µm and 4.3 µm in the short and long wavelength range, respectively, which is significantly better compared to conventional ophthalmic OCT devices [33]. Three-dimensional imaging of the eye background was performed with a fiber-coupled custom ophthalmic scanning unit [34], which facilitates easy and flexible access to the measurement area. The overall optical power of the dual-band OCT beam at the distal end of the scanning unit, i.e. on the cornea of the subject, was measured to be 780 µW, which is sufficiently low avoid light damage or photo-toxicity effects on the retina during the imaging time.

For zebrafish imaging, two custom objective lens configurations were applied in order to cover a sufficient measurement area on the retina. The first objective lens with a back focal length (BFL) of 6.8 mm enables a field of view (FOV) of approximately 8°× 8° on the retina, and was used for the diffuse light lesion measurements with almost homogeneously distributed retinal damage. The second objective lens with BFL = 3.8 mm and FOV = 30°×30° was applied for the focused light experiment, because a larger measurement area was required in order to resolve the heterogeneous spatial distribution of the lesion.

Fish were briefly anaesthesized in 0.024% Tricaine in system water until they became unresponsive to touch. Next, animals were positioned in a square plastic dish containing 0.012% Tricaine and fixed in upright position with a soft sponge. To relocate the same area in the retina during consecutive OCT imaging sessions at different time points following lesion, fish were always placed in the same position and angle. OCT imaging of the zebrafish retina was then performed through the transparent dish. The distal end of the scanning unit was positioned perpendicular to the dish and the OCT beam was aligned to the eye by means of a 3-axis linear stage assembly. To facilitate navigation in 3D OCT image data, the fast-scanning axis was oriented along the dorsal-ventral axis of the eye, thereby aligning the orthogonal slow scanning axis in anterior-posterior orientation. The alignment was visually controlled by live OCT images acquired in a crosshair scanning pattern. The easily visible blood vessels on the vitreal surface of the retina facilitated imaging consistent areas in each sample. The imaging speed was set to 12 800 axial scans (depth-resolved reflectance profiles) per second, which corresponds to 25 cross-sectional scans per second at 512 axial scans per cross-section. Finally, a three-dimensional image stack consisting of 480 cross-sections was recorded from the eye background of each animal. The entire alignment and data recording procedure took about 2 to 5 minutes. Afterwards, fish were returned to system water for recovery and kept separately. During the subsequent post-processing of OCT data, the images from the two simultaneously acquired wavelength bands were averaged in order to suppress OCT-inherent speckle noise and to guarantee maximum image quality. For time course recordings, 6 fish of the golb-1 line in AB background were imaged before treatment and at 5 distinct time points after each lesion paradigm. Additionally an untreated control sibling was imaged at every time point.

## Results

### Establishing focused light lesion method using immobilized fish

Light treatment allows the specific ablation of light sensitive cells without surgical intervention. We characterized two different light lesion paradigms using intense light in detail. One of these light lesion paradigms has been established before in normally pigmented fish [4] and compared to low intensity light lesion paradigms in albino fish [18]. Due to the undirected distribution of light in this set-up we refer to it as ‘diffuse light lesion paradigm’. The aim of the second, newly established light lesion paradigm was to direct the light onto a defined area of only one of the eyes. Accordingly, we refer to this new procedure as ‘focused-light lesion paradigm’. For both assays, we used a light source that emits UV radiation which is potentially damaging any tissue.

In case of the focused light lesion paradigm, we used a stereomicroscope to very precisely apply static illumination. Light was focused directly onto one eye of the fish using a SDF PL APO 1xPF 0.15NA objective; the other eye served as untreated control (Fig. 1A). Lens and cornea focus the light beam to a central area in the retina, allowing a targeted ablation of light sensitive cells in a specific region of the retina. We refer to this new procedure as the ‘focused light lesion paradigm’.

### Differences in light lesion properties for photoreceptor subtypes

**Figure 2:**
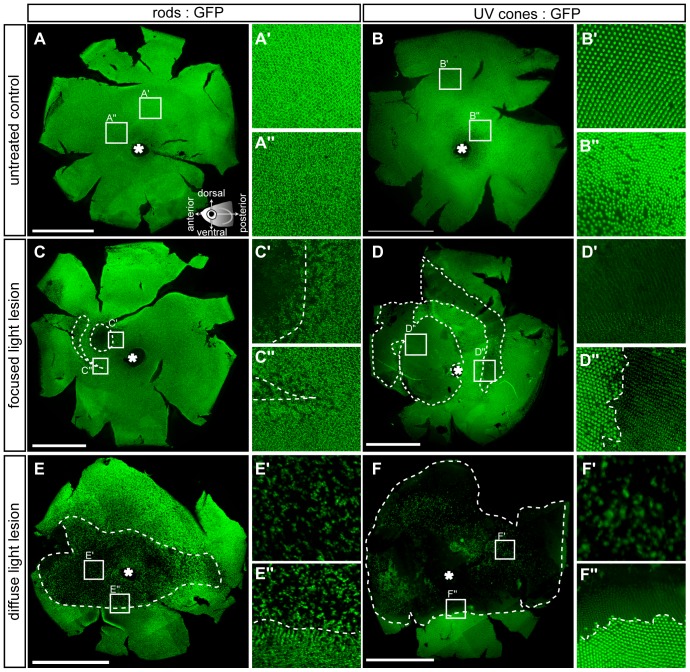
Flatmounted retina samples of lesioned GFP transgenic reporter fish. Rods (left column) are labelled with the rh1:GFP and UV cones (right column) with the opn1sw1:GFP reporter fish, respectively. **A, B:** Vitreal view of untreated control eyes. Insets show magnified images indicated in the overviews. Illustration showing the orientation of flatmounted samples as vitreal view with dorsal (superior) orientation to the top. **C-C″**: 3 days post focused light lesions in rh1:GFP. **D-D″**: 3 days post focused light lesions in opn1sw1:GFP. **E-E″:** 3 days post diffuse light lesions in rh1:GFP. **F-F″**: 3 days post diffuse light lesions in opn1sw1:GFP. The asterisk in A–F marks the optic nerve head. Scale bars represent 1 mm.

We sought to compare the already published light lesion model (Fig. 1B) and our new focused light lesion (Fig. 1A, initially with regard to lesion size. In order to get an idea of the spatial extent of damage in these two lesion paradigms, we used flatmounted retinae of the transgenic reporter lines rh1:GFP [22] and opn1sw1:GFP [23] (see Material and Methods) to visualize rods and UV cones (Fig. 2A, B).

#### Focused light lesions

After focused light lesions, two distinct areas were depleted of GFP-expressing (GFP+) photoreceptors (Fig. 2C, D). We observed a round lesion in the central retina and a semicircular lesion in a more peripheral location, both in rod and in cone reporter fish. In both lesioned areas, we observed only partial depletion of rods (Fig. 2C′). Furthermore, rods showed a gradual transition between partial depletion in the middle of a lesion area, to the regular unlesioned pattern (Fig. 2C″). In contrast, all GFP+ UV cones were completely depleted within the lesion area (Fig. 2D′). Also, UV cones displayed a sharp border between depleted cones in the light damage area, and unaffected cones outside of the lesion area (Fig. 2D″).

#### Diffuse light lesions

Diffuse light lesions elicited a large centrally located lesion that was depleted of GFP+ photoreceptors (Fig. 2E, F). The rod-depleted area was shaped as a horizontal stripe. In the peripheral retina, where only few UV cones survived, rods remained mostly undamaged. The area showing loss of rods was not completely depleted of rho:GFP+ cells (Fig. 2E′), and loss become less pronounced at the borders (Fig. 2E″). In contrast, the area of damaged UV cones was completely depleted of GFP+ cells (Fig. 2F′) with a sharp border to unaffected areas (Fig. 2F″), as observed after focused light lesion. Notably, neither of the photoreceptor subtypes was affected by light lesions in a ventral area of the retina, confirming observations from previous studies [14], [18].

**Figure 3:**
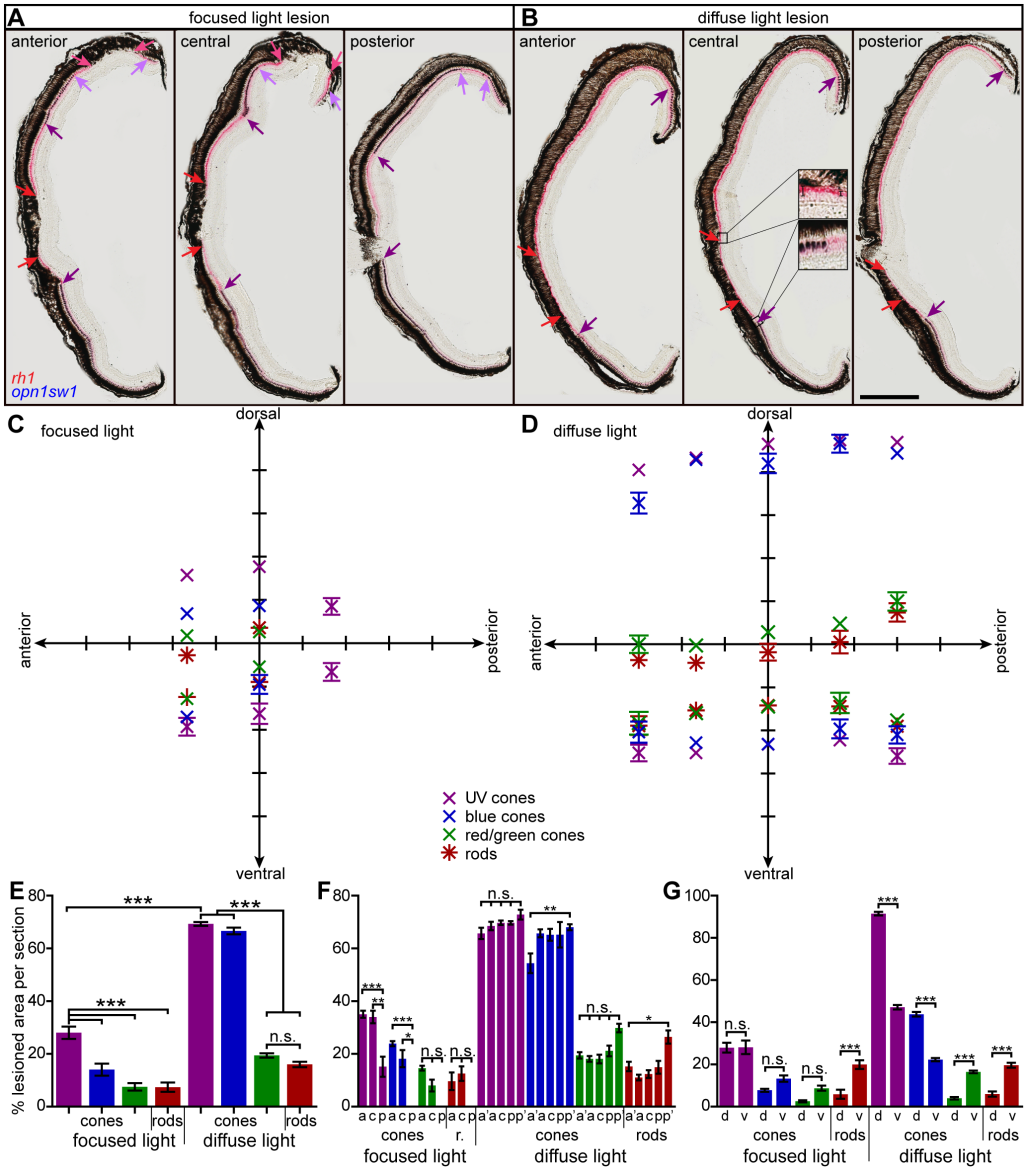
Extent of light lesioned areas in anterior-posterior and dorsal-ventral comparison. **A, B**: Double *in situ* hybridization against *rhodopsin* (red) to label rods and *opn1sw1* (blue) to label UV cones. **A:** Focused light lesioned retinas in an anterior (left), central (middle) and posterior section (right). Central UV cone lesions are labeled with violet arrows, rod lesions are labeled with red arrows. Light purple and pink arrows indicate peripheral lesions of UV cones and rods, respectively. **B:** Diffuse light lesions analogous to A. Insets in the central section show the transition from healthy to lesioned areas. **C:** Blot of lesioned areas relative to their location in the retina. The average of dorsal and ventral lesion boundaries are blotted in the respective color code indicated in the legend. **D:** Lesioned areas analogous to C for diffuse light paradigm with additional far anterior and far posterior measure points. **E:** Comparison of average total damaged area in % of total retina length. **F:** Comparison of average anterior vs. posterior damaged area in % of total retina length. **G:** Comparison of average dorsal vs. ventral damaged area in % of 1/2 retina length. a: anterior, a′: far anterior, c: central, d: dorsal, p: posterior, p′: far posterior, r: rods; v: ventral. Column colors indicate cone type as in the legend (C). Error bars indicate standard error of the mean; *** for p-values <0.001; ** for p-values <0.01; * for p<0.05; n.s.: not significant; scale bar represents 400 µm.

Based on the initial observations from the transgenic reporter fish, we subsequently determined the exact spatial extent of light lesions with double *in situ* hybridization on transversal sections of adult zebrafish at 3 dpl (Fig. 3). To avoid common problems such as GFP photo bleaching or photo toxicity in transgenic GFP reporter lines, we used WT fish instead [35]. Measurements of lesioned areas were performed in a quantitative and objective manner for each photoreceptor subtype by specific probes binding to the respective opsin mRNA. In anterior and central sections of focused light lesions, the central retina is depleted of rods (Fig. 3A, red arrows) and enclosed by UV cone depleted areas (Fig. 3A, violet arrows). The peripheral lesion (Fig. 3A, light purple and pink arrows) was not included in measurements because of high variability in its presence and extent. In the case of diffuse light lesions the extent of the central lesion is determined in the same manner (Fig. 3B, arrows). We measured the lesion size for photoreceptor subtype as percentage of the retinal length in each respective retina section (for detailed description see material and methods) and visualized the results as average ventral and dorsal lesion border along the anterior-posterior axis (Fig. 3C, D). Comparison of the average total lesioned area per retinal section (Fig. 3E, Table 2) confirms that the damaged area is larger after diffuse light lesions than centrally after focused light lesions in case of each individual photoreceptor subtype, respectively. In focused light lesions, the UV cone depleted area is largest compared to the other photoreceptor subtypes. In a similar tendency, the area depleted of UV and blue cones in diffuse light lesions is at least three-fold larger than the size of red/green cone and rod depleted areas indicating different susceptibilities to the light source corresponding to the light sensitivity of the respective opsin. Moreover, the percentage of depleted retina area follows a gradient corresponding to specific wavelengths with small lesions in the long- and middle wavelength range (rods and cones), intermediate blue cone lesions and largest light lesions in short wavelength range (UV cones).

**Table 2:** Total average photoreceptor lesion extents ± SEM in percent of total retina length.

	focused light lesion	diffuse light lesion
UV cones	28.05±2.34	69.30±0.71
blue cones	14.02±2.27	66.62±1.27
red/green double cones	7.53±1.40	19.42±0.85
rods	7.39±1.74	16.00±1.02

Previous work has indicated differences in anterior-posterior and dorsal-ventral susceptibility depending on the light damage paradigm. In brief, the posterior retina seems more susceptible than the anterior retina whenever long-term treatment with low light intensity is involved. In regard to the dorsal-ventral axis, all studies agree that the area ventral of the optic nerve head is least susceptible whereas light damage can always be found dorsal from the optic nerve head [4], [14], [18]. To evaluate whether different photoreceptor subtypes show differences in regard to anterior-posterior susceptibilities in our lesion paradigms, we compared the average lesion size along the anterior-posterior axis for each individual photoreceptor subtype (Fig. 3F, Table 3). This analysis has revealed that after focused light lesions, the damaged area increases along the posterior- anterior axis in case of UV cones and blues cones –the photoreceptors subtypes that are affected strongest after lesion as shown above. This result can be explained by the anatomy of the fish eye, which is tilted towards anterior in the laterally placed fish. In diffuse light lesions all photoreceptor subtypes show a relatively homogeneous size of lesion along the anterior-posterior axis within the same area analysed in case of focused light lesion (Fig. 3F, diffuse light lesion, column a, c, p). To investigate whether we observe differences in anterior-posterior susceptibly when we extend the analysed area along the anterior-posterior axis, we measured more peripheral lesions and compared the far anterior and far posterior sections. Here, we find a significantly bigger posterior lesion in blue cones and rods only (Fig. 3F, diffuse light lesion, column a′, p′). In conclusion, in focused light lesion posterior photoreceptors were spared of lesion whereas rods and blue cones are slightly spared in the anterior diffuse light lesions.

**Table 3:** Average anterior-posterior photoreceptor lesion extents ± SEM in percent of total retina length.

	focused light lesion				diffuse light lesion			
	anterior	central	posterior	far anterior	anterior	central	posterior	far posterior
UV cones	35.02±1.33	33.97±2.37	15.16±3.83	65.71±2.11	67.78±1.51	69.67±0.87	69.81±0.71	72.85±1.86
blue cones	23.87±0.97	18.2±3.26	0±0	54.37±3.72	65.61±1.50	65.17±2.29	65.22±4.84	68.02±1.15
red/green double cones	14.57±0.90	8.02±2.24	0±0	19.42±1.19	18.08±1.08	18.09±1.63	21.14±2.02	29.82±1.63
rods	9.63±3.33	12.55±2.77	0±0	15.23±1.81	11.19±1.1	11.88±1.48	13.7±2.21	26.42± 2.51

Also, along the dorsal-ventral axis, we observed photoreceptor-subtype specific lesion patterns (Fig. 3D, G, Table 4). The focused light lesion is located centrally for all cone subtypes, whereas the rod lesion covers 3 times more of the ventral than of the dorsal half of the retina (Fig. 3G). In contrast, after diffuse light lesion, the size of the dorsal lesion exceeds the size of the ventral lesions by factor two in case of the short wavelength sensitive cones (UV and blue). The mid and long wavelength sensitive photoreceptors instead (red/green double cones and rods) are damaged within a much smaller area and the size of their ventral lesion is four-fold larger than of their dorsal lesion (Fig. 3B, G).

**Table 4:** Average dorsal-ventral photoreceptor lesion extents ± SEM in percent of half retina length.

	focused light lesion		diffuse light lesion	
	dorsal	ventral	dorsal	ventral
UV cones	27.94±2.32	28.16±3.19	91.47±1.11	47.13±1.11
blue cones	7.75±0.73	13.23±1.52	43.76±1.10	22.30±0.74
red/green double cones	2.58±0.50	8.72±1.22	3.95±0.58	16.49±0.66
rods	5.90±2.18	19.95±2.04	5.94±1.26	19.63±1.20

**Figure 4:**
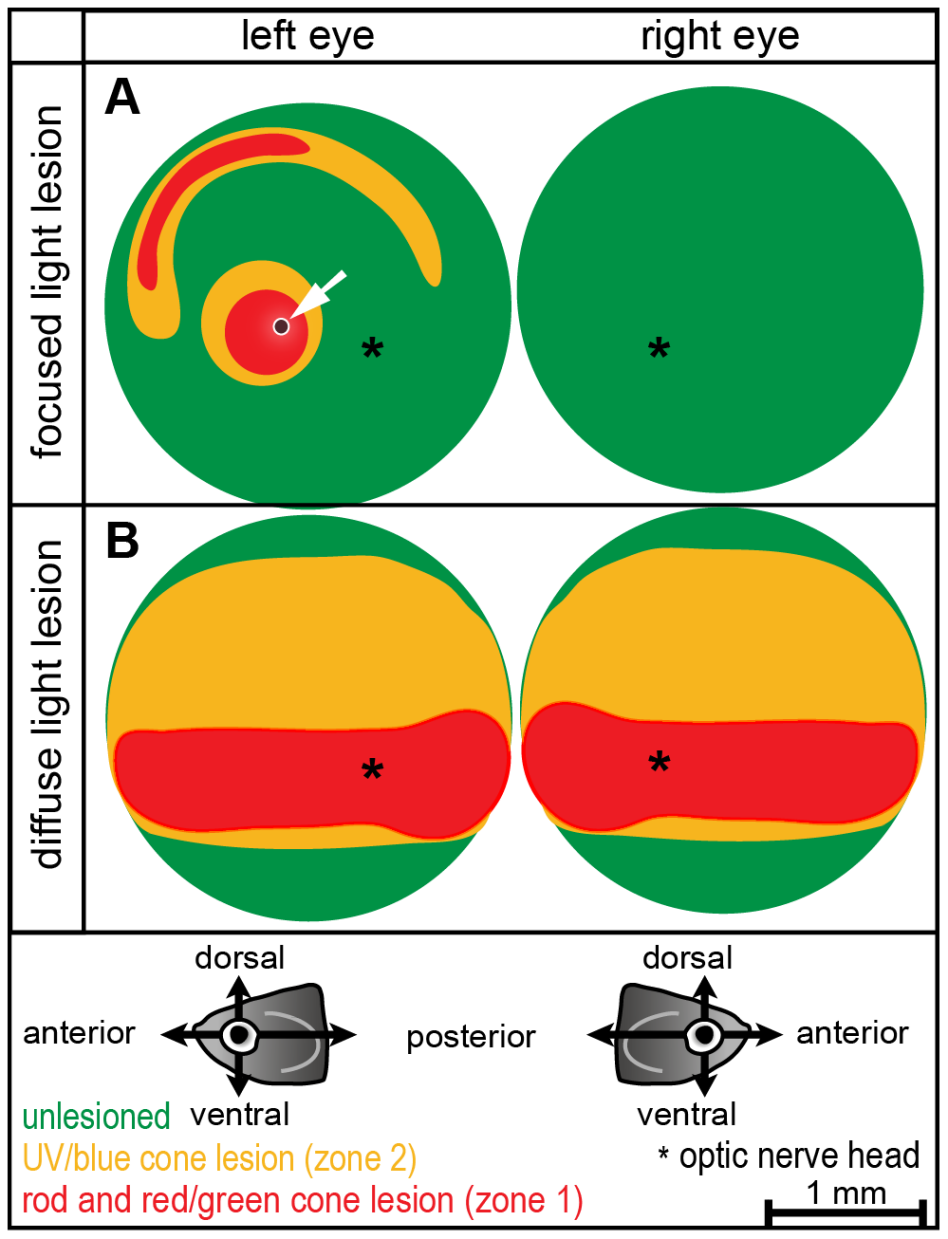
Schematic illustration of lesion patterns. **A**: Focused light lesions form a complex lesion pattern in the central and peripheral retina. A small area of unspecific damage in all retinal layers is due to the high intensity of the center beam, and is indicated by the white arrow. Most of the light treated retina and the control eye on the other side is undamaged (green). **B:** Diffuse light lesions in the central retina are shaped as horizontal stripe. Around the area in the center (depleted of all photoreceptors) are UV and blue cone depleted areas in the peripheral retina (yellow). The ventral part is never damaged in any fish. (Orientation as shown in illustrations below; asterisk represents optic nerve head).

In conclusion, we can describe two lesion zones as summarized in Figure 4: Zone 1 is characterized by loss of all photoreceptor subtypes and located centrally. Zone 2 is depleted of the short wavelength light sensitive blue and UV cones only and encloses zone 1. After diffuse light lesions, zone 1 is located along the midline of the retina within a horizontal band that comprises around 17% of the retinal area (Fig. 4B). Zone 2 extends over about 50% of the total retina area and more than two third (73%) of it locate in the dorsal half in diffuse light lesion. In focused light lesions, both zones are round and located close to the centre of the retina (Fig. 4A). Similar to diffuse light lesions, the small central zone 1 comprising 7.5% of retina area is enclosed by a bigger zone 2 extending over 14% of retina area.

In both paradigms, the ventral retina is largely spared of damage in agreement with previous studies [4], [18]. The susceptibility of different photoreceptor subtypes correlates with their specificity of wavelength sensitivity, indicating that the UV emitting light source is a possible reason for the strong effect of diffuse light lesions onto UV and blue cones. Moreover, the morphology of photoreceptors and their state of contraction in the light adapted retina [36] as well as their contact to neighbouring RPE cells might influence the susceptibility of the different photoreceptor subtypes. In previous work on diffuse light lesions, cell loss per 300 µm retina length was determined by counting cell bodies in transversal sections containing the optic nerve [18]. In contrast to our results, the percentage of cell loss was higher for rods than for cones and more cells were lost in the dorsal half than in the ventral half of the retina. To compare our results about regional specificity of lesions and the photoreceptor subtype specific susceptibility received in normally pigmented fish with the above mentioned data published in albino fish, we quantified absolute cell numbers (Table 5) in the same manner as described by Thomas et al. However, to label rod and cones, we did not perform immunohistochemical stainings but *in situ* hybridization as the published antibodies are not commercially available. The optic nerve head locates in the ventral half of our sections and does not divide the section in two equal halves. In the following, we will refer to the counting area dorsal of the optic nerve head as dorsal counting area although it is not completely within the mathematically determined dorsal half of the retina. Analogous, the area ventral from the optic nerve head is referred to as ventral counting area and it should be pointed out that this area is more peripheral than the dorsal counting area. In summary, we observed a 31.4% reduction in number of rod cell bodies in the dorsal, but none in the ventral counting area. In the case of UV cones we find a 98% reduction of cell bodies on the dorsal, but only 4% on the ventral counting area, consistent with our findings in lesion patterns where zone 2 locates primarily dorsal. Within the limited counting areas, the percentage of cell loss in UV cones predominates over rods, although the absolute loss of cells is higher in rods (on average 72 rods vs. 43 UV cones) due to the different densities of the photoreceptor subtypes. Taken together we can conclude that in our model at least UV cones are indeed more sensitive to light damage than rods. In this context it is also important to point out that the number of rod cell bodies in the untreated albino retina already seems to be reduced by roughly 50–70% compared to our untreated WT AB retina, whereas the UV cone density is similar in the two different samples (i.e. in the dorsal counting area; rods: 81.4±1.9 (albino) vs. 230.5±21.1 (WT AB); UV cones: 45.6±2.3 (albino) vs. 43.6±2.3 (WT AB)). The main difference between the two models is the use of differently pigmented fish strains for experiments. The albino fish lack melanin granules in the Retinal Pigment Epithelium (RPE) and are therefore not light protected as discussed above.

**Table 5:** Average number of photoreceptor cell bodies per 300 µm retina length ± SEM after light lesion and respective P-value for control vs. 3dpl comparisons.

	control	3 dpl	P-value
Rods, Dorsal	230.5±21.1	158.1±8.5	0.0161
Rods, Ventral	152.4±9,5	159.5±5.4	0.5568
UV cones, Dorsal	43.6±2.3	0.4±0.4	4.59E-09
UV cones, Ventral	49.8±1.7	48.2±1.5	0.5029

In summary, focused light lesion depletes photoreceptors both in central and in peripheral regions of the retina. Diffuse light lesion depletes photoreceptors only in a central area, which is however larger than after focused light lesion. Within each light lesion, two lesion zones are determined by ablation or at least reduction of all photoreceptor subtypes (zone 1) or only blue- and UV-sensitive cones (zone 2) corresponding to the different wavelength sensitivity of photoreceptors (summarized in Fig. 4).

### Optical properties of the fish eye lead to a distinct focused light lesion pattern

**Figure 5:**
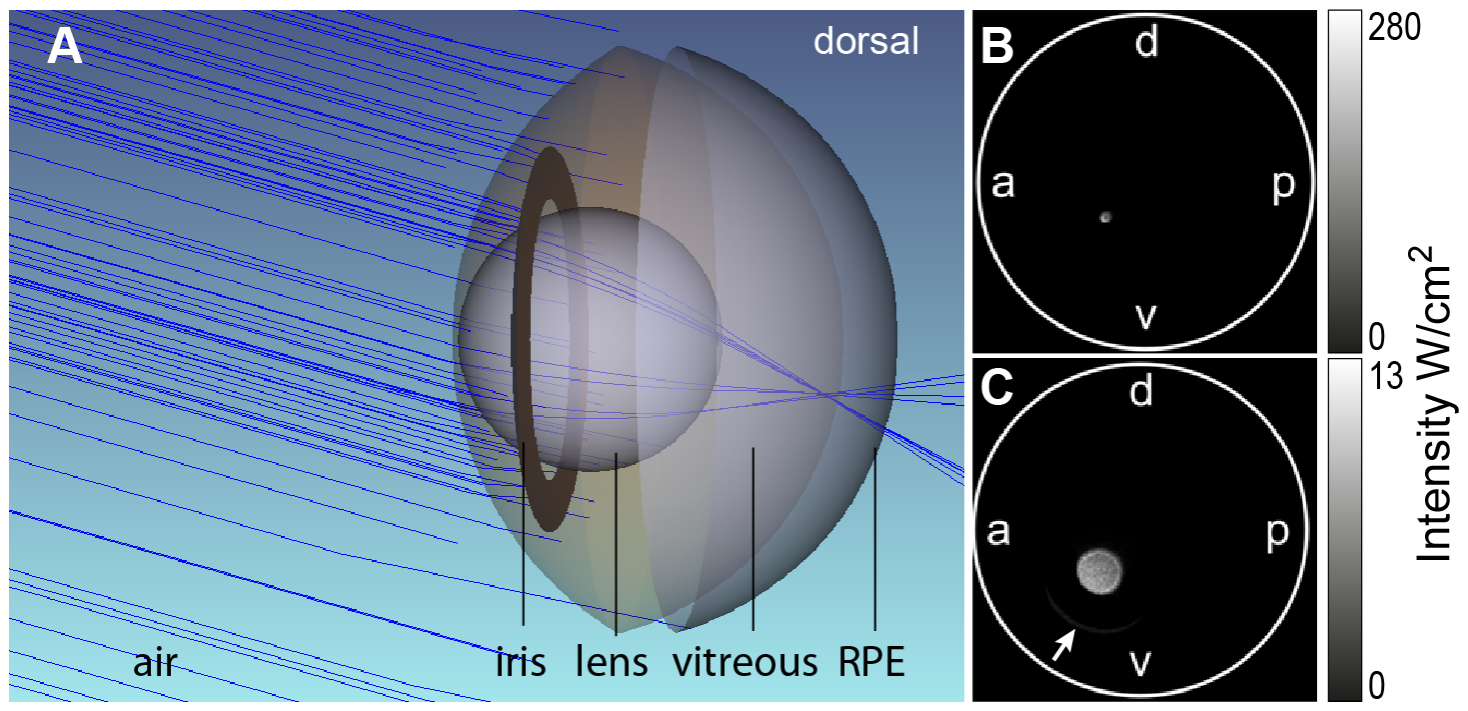
Model of focused light exposure. **A**: Simulation of light beams (blue lines) in focused light lesions. Most light is refracted by the lens to a central spot in the retina. Photoreceptors close to the RPE (grey) are not in the focus of light beams. **B:** Light detection at the approximate position of the retinal nerve fiber layer in Zemax© fish eye model (flattened top view). **C:** Light detection at the approximate position of the RPE. A small gap in between iris and pupil allows a little light to pass directly, causing a small sickle below the lesion center (arrow). The eye was tilted relative to the light beam by 18.3° towards anterior and 15.8° in dorsal direction to simulate the conditions in focused light lesion.

To explain the lesion induced by the focused light lesion, we developed an optical model to simulate the course of light beams within the eye. Light enters the eye through the cornea and passes the pupil. Then, a spherical lens refracts the light to the central retina. In focused light lesions, only a small area of the retina is constantly exposed to light, resulting in a small area of damage compared to diffuse light lesions. Simulation of the fish eye as a simplified optical system in the Zemax software (Fig. 5A) enables also detection of light in the eye background. The location of the focused lesion in the retina center was confirmed in our model (Fig. 5B, C). The tilted zebrafish eye and light beam were represented by tilting the eye model relative to the light source. As a result, the typical lesion was formed outside of the retina center, as observed also experimentally. We detected high energy in the focus near the ganglion cell layer (GCL), which perfectly matches a small area of unspecific damage (Fig. 5B). Additionally, some light passes directly through a gap between lens and iris resulting in an increased area of photoreceptors hit by lower light intensity (Fig. 5C, arrows). This affects therefore mainly photoreceptors which are more prone to light damage (see Fig. 2D) In short, we established an optical model of the fish eye that can simulate the path of light beam and allows us to evaluate its relative intensity in different areas of the retina.

### Cell death occurs in distinct time frames in most cells of the outer nuclear layer

**Figure 6:**
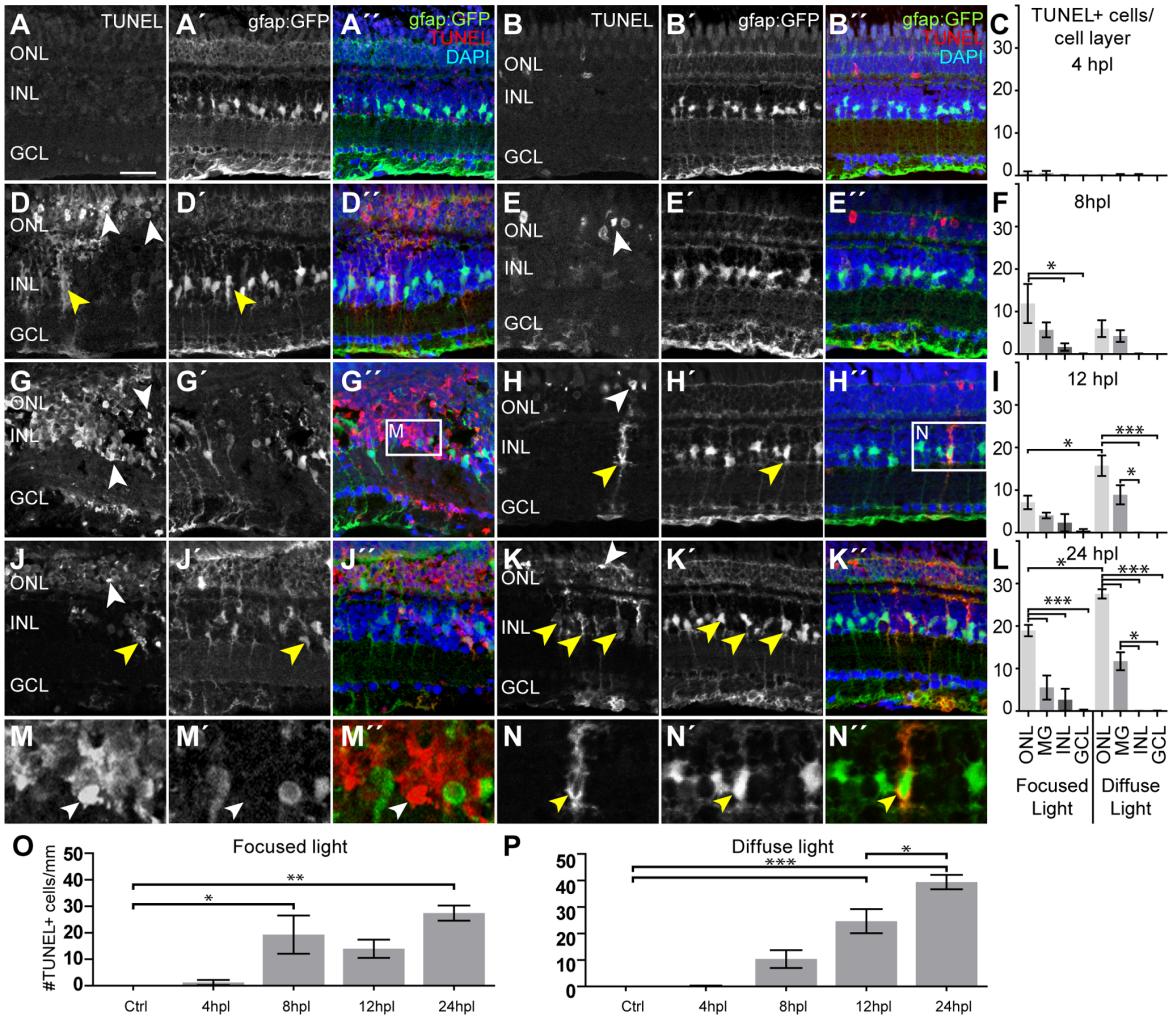
Cell death after light lesions in Tg(gfap:GFP) fish. TUNEL+ cells are labeled in red, MG are GFP+ (green), nuclei are stained with DAPI (blue). Quantification shows TUNEL+ cells per cell layer normalized to 1 mm retinal length in (C, F, I, L). GFP/TUNEL double positive cells are indicated as ‘MG’ to discriminate between phagocytic MG and real INL cell death. **A–C:** Only very few TUNEL+ cells were found early at 4 hpl. **D–I**: Increasing amounts of TUNEL+ cells were found between 8 hpl and 12 hpl (white arrowheads). The density of TUNEL+ cells is high in the center of focused light lesions but lower along the whole retina after diffuse light lesions. **J–L:** A peak of TUNEL+ cells is found at 24 hpl in both lesion paradigms. **M:** Many TUNEL+ INL cells that are GFP- (white arrowhead) were found in the centre of focused light lesions, but were rare elsewhere. **N:** Example of TUNEL+ MG. TUNEL+ signal is found in the cytoplasm (yellow arrowhead). **O, P:** Time course of the total number of TUNEL+ cells per mm peaking at 24hpl. dpl: days post lesion; GCL: ganglion cell layer; hpl: hours post lesion; INL: inner nuclear layer; MG: Müller glia; ONL: outer nuclear layer; Error bars indicate standard error of the mean; *** for p-values <0.001; ** for p-values <0.01; * for p<0.05; scale bars represent 20 µm.

Light lesion is thought to cause cell death in the illuminated retinal areas [14], [18], [37]. To study the kinetics and specificity of cell death in the different light lesion paradigms, we labeled dying cells and monitored the time course of cell death by TUNEL staining (Fig. 6, Table 6). At 4 hours after both lesion paradigms, the retina was still largely devoid of TUNEL staining (Fig. 6A–C). Between 8 and 12 hours post lesion (hpl), the overall number of TUNEL+ cells significantly increased after both lesion paradigms (Fig. 6D–I, O, P). Although initiation of cell death was apparent in both lesion paradigms at 8 hpl, more TUNEL+ cells were found in distinct clusters after focused light lesions, indicating an earlier onset of cell death in the severely damaged areas. Separate analysis of TUNEL+ cells in the different retinal layers revealed that the majority of TUNEL+ cells mainly locate within the ONL at all analyzed time points (Fig. 6F, I, L). The density of TUNEL+ cells reflects the severity of damage: Whereas the small focused light lesion showed big central clusters of TUNEL+ cells, only single TUNEL+ cells were homogeneously distributed across diffuse light lesioned areas (Fig. 6 D, E, white arrowheads). Following focused light lesion, we observed TUNEL+/GFP− cells in the inner nuclear layer (INL) of the central focused light lesion, indicating a locally confined area where unspecific cell death occurs to other cell types than photoreceptors (Fig. 6G, M). Within the same area, also in the GCL a low number of TUNEL+ cells was found (Fig. 6I, L; Table 6). For both paradigms the number of TUNEL+ cells peaks at 24 hpl (Fig. 6J–L, O, P) however we observe differences in the kinetics, namely fast onset with two small peaks of TUNEL+ cells after focused light lesion in contrast to a gradually increasing number of TUNEL+ cells after diffuse light lesions peaking at 24 hpl.

**Table 6:** Average number of TUNEL positive cells per mm retinal length ± SEM during degeneration of light lesioned retinae.

	focused light lesion				diffuse light lesion			
	ONL	MG	INL	GCL	ONL	MG	INL	GCL
4 hpl	0.54±0.49	0.60±0.51	0.06±0.06	0.03±0.03	0.03±0.03	0.21±0.13	0.21±0.13	0±0
8 hpl	11.88±4.59	5.70±1.76	1.66±0.87	0.083±0.083	6.00±2.00	4.25±1.38	0.08±0.08	0±0
12 hpl	7.11±1.58	4.02±0.69	2.33±2.05	0.5±0.35	15.73±2.42	8.9±2.25	0.03±0.03	0±0
24 hpl	18.95±1.33	5.55±2.85	2.67±2.59	0.25±0.1	27.59±1.1	11.73±2.11	0.03±0.03	0.06±0.06

Previous reports indicated that MG as well as invading microglia participate in removing cell debris following lesion of the retina [4], [17], [38]–[40]. To characterize the abundance of these to cell types after lesion, we performed stainings for the leukocyte marker L-Plastin and cell death in the gfap:GFP fish labeling MG. After both lesion paradigms, TUNEL+/GFP+ MG were found between 8 hpl and 24 hpl (Fig. 6D–N, yellow arrowheads). We found no TUNEL staining in the nucleus of these MG (Fig. 6N), indicating that they are not dying themselves, but rather phagocytose dead cells, in agreement with previous reports [39]. In the small focused light lesions the number of phagocytic MG does not change over time although cell death in ONL and INL increase until 24 hpl (Fig. 6 F, I, L), which could be due to the locally confined lesion that does not allow more MG to participate in the phagocytic processes. After diffuse light lesion at least 99.7% of TUNEL+ cells in the INL were GFP+ MG (Fig. 6H, N). Between 8 hpl and 12 hpl the two-fold increase in TUNEL+ cells in the ONL was accompanied by a two-fold increase of phagocytic MG. Similarly, between 12 hpl and 24 hpl the number of TUNEL+ ONL cell doubled again and the number phagocytic MG also increased by 1,3-fold, indicating that cell death gradually spreads across all the lesioned area between 8 hpl and 24 hpl.

The leukocyte marker L-Plastin seldom co-localized with TUNEL+ cells, but was often found closely adjacent to them (Fig. S1). This proximity of TUNEL+ cells to macrophages has been found *in vitro* in previously published work as an indication for uptake of dead cells [41]. Furthermore, L-Plastin+ cells did not co-localize with TUNEL+ MG in the INL. Quantification of the total number of L-Plastin+ cells per mm retina length shows an immediate increase at 4 hpl (before cell death initiated), a decline between 4 hpl and 12 hpl and a second and a peak at 24 hpl correlating with the peak of cell death (Fig. S1C, D) On average, the total number of L-Plastin+ cells found in diffuse light lesions was 1.4-fold higher compared to focused light lesions and the difference in L-Plastin+ cell numbers between control and 12 hpl is significant for diffuse light only. However, a similar trend was observed in focused light lesions, although the lesioned area is significantly smaller.

In summary, we observed an early onset of cell death that peaks at 24 hpl. After focused light lesions cell death initiates early at 8 hpl with an additional first peak, whereas a gradual increase of TUNEL+ cell was found after diffuse light lesions. Most cell death was found in the ONL at each analysed time point (Fig. 6F, I, L), but a few TUNEL+ cells were also found in the INL following focused light lesions. Because dead cells can be detected only within a distinct time window, we conclude that these two lesion models initiate cell death in similar time frame in all affected cells. Our data suggest that not only MG but also leucocytes remove cell debris after light lesion without one mechanism predominating in one of the paradigms.

### Differential removal of UV cones in zone 1 and 2

**Figure 7:**
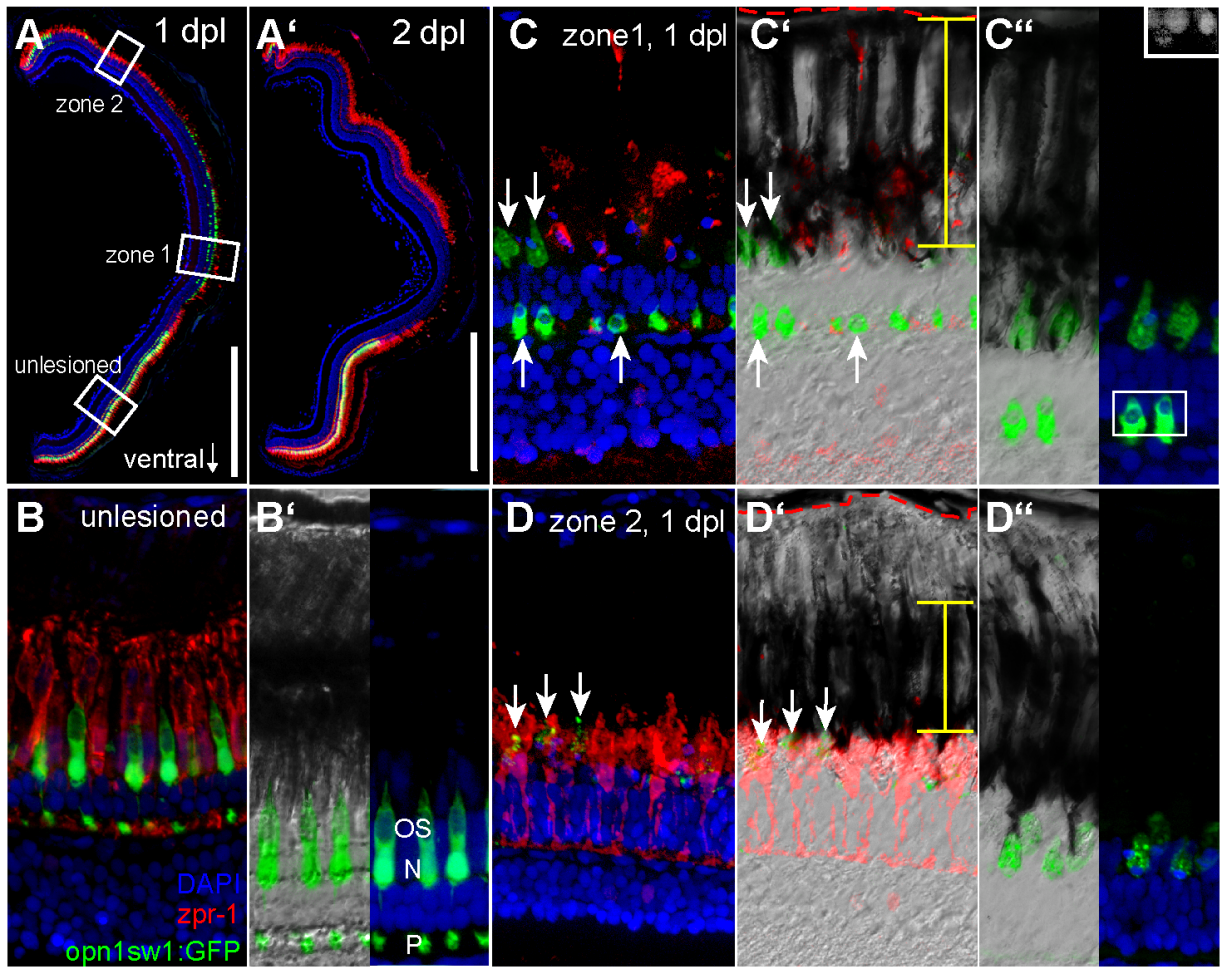
Differential removal of dying UV cones in zone 1 and 2. **A**: Overview image of a retinal section from an opn1sw1:GFP transgenic animal at 1 and 2 days post diffuse light lesion (**A′**). **B**: Unlesioned red/green double cones are labelled by zpr-1 (red) and UV cones by GFP. Nuclei were stained with DAPI (blue). **B′:** Close up of outer segments (OS), nucleus (N) and pedicle (P) is in the OPL. **C:** Central retina (zone 1) showing removal of zpr1+ cones while disrupted UV cones persist (arrow). **C′:** DIC image showing the distribution of pigmented granula in the RPE (yellow bar) relative to Bruch's membrane (red dashed line). **C″:** Inset shows pyknotic nuclei in DAPI channel. **D:** Peripheral lesion with intact red/green cones but depleted UV cones. **D′:** DIC image showing condensed RPE pigments. **D″:** Co-localisation of pigmented processes with remaining GFP debris. ONL: Outer Nuclear Layer; RPE: Retina Pigmented Epithelium. Scale bars represent 500 µm.

Light lesions lead to cell death of photoreceptors already at 1 dpl. From a detailed time course analysis of the photoreceptor reporter lines opn1sw1:GFP and rh1:GFP however, we know that the cell debris of dead photoreceptors is not removed completely from the retina until 3 dpl (data not shown). We found differential removal of photoreceptors in correlation to different RPE morphologies at 1 dpl and 2 dpl after diffuse light lesion. The Zpr-1+ red/green double cones and GFP+ UV cones are not equally affected in the lesion zones (Fig. 7A). At 2 dpl zone 1 is clearly depleted of Zpr-1+ cells and UC cones but zone 2 is only depleted of GFP+ UV cones. In the unlesioned ventral retina, Zpr-1+ cones have longer outer segments (OS) than the GFP+ UV cones (Fig. 7B). Within the diffuse light lesion the morphology of the Zpr-1+ long double cones in zone 1 was completely disrupted at 1 dpl (Fig. 7C, red). Within the ONL, pedicles, inner fibers and cell bodies of Zpr-1+ cells were completely cleared away. We detected remaining Zpr-1+ cell debris only within or next to pigmented RPE cells. The morphology of the UV cones was also severely affected (Fig. 7C, green). In contrast to Zpr-1+ cells, a portion of the inner segments of the UV cones remained within the ONL as strongly GFP+ debris containing pyknotic nuclei (Fig. 7C″, inset). Apical processes of the RPE cells co-localize with Zpr-1+ debris as well as GFP+ debris in retinal regions basal to the ONL, but not with pyknotic GFP+ nuclei within the ONL (Fig. 7D).

At the same time, in zone 2 we observed depleted GFP+ UV cones, but intact Zpr-1+ cones (Fig. 7E). Processes of the RPE cells stretch more apical than in zone 1 towards UV cones close to the ONL and enclose them (Fig. 7E′, arrows). Within this area, we observed less GFP+ debris compared to zone 1, suggesting that the RPE processes will eventually clear all GFP+ debris. Our data indicate that the difference between clearance of photoreceptor debris correlates with different morphologies of RPE cells. The basal membrane of the RPE, called Bruch's membrane (red dashed line), represents the basal limit of the retina. Within zone 1 (Fig. 7C′), where both cone types were affected, pigmented granules of RPE cells (yellow error bar) were spread between the Bruch's membrane and photoreceptor OS. However, in zone 2, where red/green double cones were intact, we found the pigmented granules condensed at a distance from the Bruch's membrane (Fig. 7E′). Little attention has been paid to a potential role of retinal pigmented epithelium (RPE) cells after acute light damage. The apical processes of RPE cells phagocytose outer segments (OS) of shed cone discs during photoreceptor renewal in the unlesioned retina (for review see [42], [43]). Our data suggest that the RPE might also play a role in removing dead photoreceptors during regeneration, in addition to Müller glia cells and macrophages. The observed morphological differences between central and peripheral RPE cells suggest, that these cells remove nearby cell debris first, before elongating towards farther cell debris. In case they encounter less cell debris from long OS, their processes seem to elongate faster towards short OS cell debris.

### Reactive proliferation after light lesions

**Figure 8:**
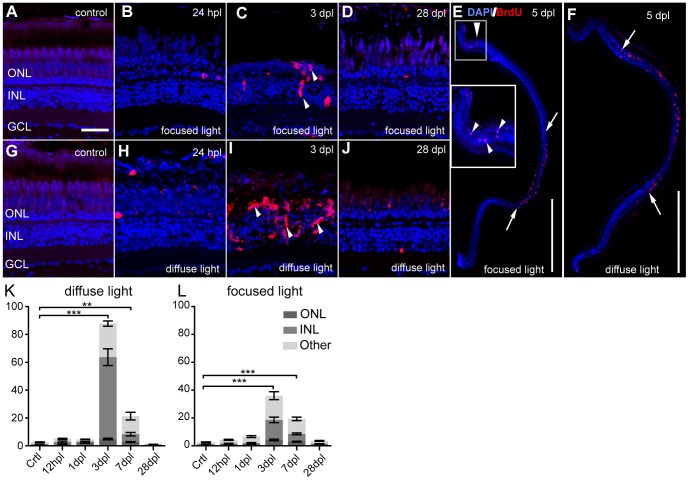
Proliferative response after light lesions. Proliferating cells are labelled by BrdU (red). **A, G**: Untreated control shows weak autofluorescence in photoreceptors. **B–D**: Clusters of BrdU+ cells are indicated by white arrowheads. **E–F:** Overview images showing proliferating cells at 5 dpl in the characteristic lesion areas (between arrows). **G–J**: Time course after a diffuse light lesion. **K, L**: Quantification of BrdU+ cells in the whole retina per cell layer normalized to 1 mm retinal length. Most proliferating cells are found in the INL and ONL at 3 dpl. BrdU+ cells found in the ganglion cell layer and the ciliary marginal zones are indicated as ‘Other’. Error bars indicate SEM. Scale bar represents 20 µm (A–D, G–J) or 500 µm (E, F).

Regeneration in the adult telencephalon of zebrafish is initiated in response to stab wound lesions through reactive proliferation of endogenous stem cells to generate proliferating multipotent progenitors [25]. We wanted to determine if light lesions cause a similar induction of MGC progenitors, and therefore assessed the number of BrdU incorporating cells at various time points after the lesion by a short pulse prior to sacrificing the fish (Table 7). In untreated retinae BrdU+ cells are rarely found (Fig. 8A, G, K). BrdU incorporation was first observed at 24 hpl (Fig. 8B, H) and peaked at 3 dpl (Fig. 8C, I) before it reached control levels again at 28 dpl (28 dpl focused light: 9.5±4.9; Ctrl: 7.2±3.44; n = 4; Fig. 8D, J). There are significantly more BrdU-positive cells in diffuse light lesions compared to focused light lesions (Fig. 8K, L). BrdU incorporating cells are only found within the characteristic lesion areas in the central and peripheral retina for focused (Fig. 8E, between arrows) and broad central in diffuse light lesions (Fig. 8F).

**Table 7:** Average number of BrdU incorporating cells per mm retinal length ± SEM during regeneration in light lesioned retinae.

time point	focused light	diffuse light
Ctrl	2.7±0.29	
12 hpl	4.36±0.53	5.11±0.71
24 hpl	6.64±3.97	4.64±0.74
3 dpl	36.07±3.97	87.85±7.75
7 dpl	19.35±1.95	21.32±4.08
28 hpl	3.55±0.42	1.08±0.20

In summary, regeneration starts in the lesioned retina with a characteristic peak of BrdU incorporation after both lesion paradigms, confirming previously published results [4], [10], [19].

### Müller glia in light lesions

**Figure 9:**
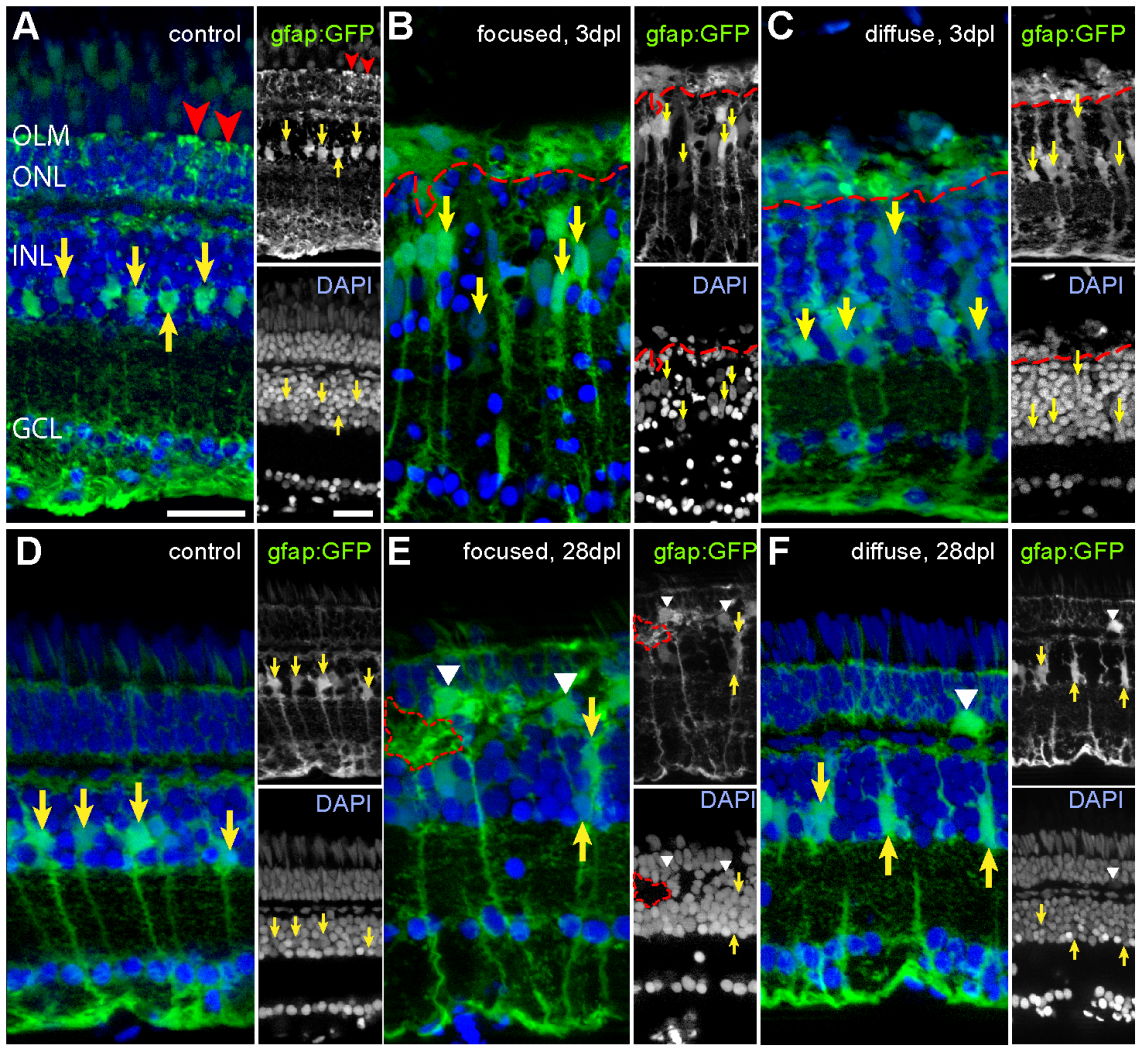
Müller glia cells after light lesions, labelled by the gfap:GFP reporter line (green) and DAPI (blue). Somata and processes of MG are indicated by yellow and red arrows, respectively. **A:** Untreated control retina. MG processes in the ONL are indicated by red arrowheads. **B:** 3 days after focused light lesion in the lesion centre. Swollen MG nuclei were found (yellow arrows). MG processes in the ONL collapsed (above dashed red line). **C:** 3 days after diffuse light lesions. ONL processes of MG collapsed (above dashed red line). **D:** Control side of focused light treated fish after 28 dpl. **E:** 28 days after focused light lesion in the lesion centre. Somata of MG are larger and sometimes displaced to the ONL (white arrowhead). Gaps in DAPI channel (dashed red line) are filled with GFP+ MG. **F:** 28 dpl after diffuse light lesion. Some displaced MG were found in the ONL (white arrowhead).

MG are the major source of progenitor cells in retina regeneration [6], therefore it is essential to understand their characteristics in the two different lesion paradigms. We used heterozygous animals of the transgenic reporter line Tg(gfap:GFP)^∧^mi2001 [15] for quantification and morphological analysis of MG after light lesions. In the unlesioned retina the processes of MG extend from the outer limiting membrane to the vasculature on the vitral surface of the retina [44]. Their somata are located in the INL close to the inner plexiform layer (IPL) (Fig. 9A, arrows). Morphology drastically changed at 3 dpl in the centre of focused light lesions. The nuclear layer structure of the retina was disrupted and MG somata appeared misarranged and swollen (Fig. 9B). Also, the nuclei of the MG were swollen, whereas the nuclei of other retinal cells retained normal appearance (see inset, arrows). Furthermore, the MG processes, formerly wrapping around inner segments of photoreceptors (Fig. 9A, red arrowheads), collapsed after both light lesion paradigms and appeared as diffuse GFP+ cell mass in the remnant of the ONL (above dashed red line).

In zone 1 and 2 of diffuse light lesions as well as outside the centre of focused light lesions, MG display similar but less drastic changes in morphology. Here the somata of MG were only slightly swollen (Fig. 9C). The MG nuclei appeared normal size as in the control (Fig. 9C, arrows).

At 28 dpl after focused light lesion the morphology of MG still showed some differences compared to untreated controls. GFP+ somata and processes of MG appeared enlarged (Fig. 9E, arrows). Misarrangements or gaps in the regular retinal layers observed in DAPI stained sections are filled with these GFP+ cell processes of MG (Fig. 9E, red dashed line). Furthermore, we found atypical GFP+ cells within the ONL (Fig. 9E, white arrowheads). After diffuse light lesions these effects were milder, but we also found swollen MG and misplaced GFP+ cells in the ONL (Fig. 9F, arrowhead). In summary, the numbers of MG in light lesions did not highly differ at any time compared to controls (Fig. S2), but their morphology is altered during regeneration.

### Regeneration is completed within one month

**Figure 10:**
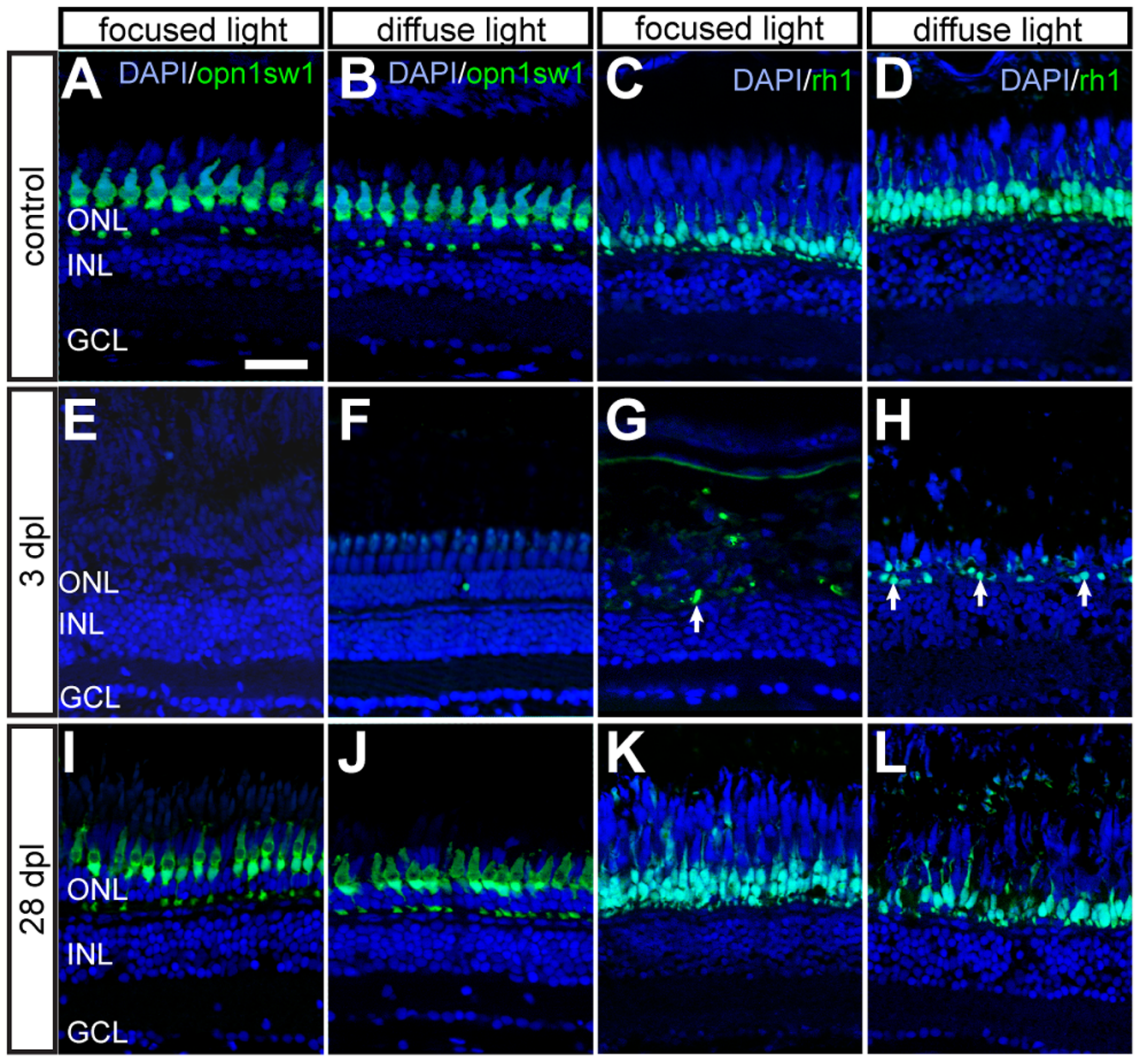
Time course of GFP+ photoreceptors after light lesions. UV cones (left panel) and rods (right panel) are labelled by the opn1sw1:GFP and rh1:GFP reporter line, respectively. **A, B**: Untreated control showing continuous mosaic pattern of UV cones. **C, D:** Untreated control showing the continuous rod layer in the ONL. **E, F:** Loss of all GFP+ cones 3 days after focused and diffuse light lesions. **G, H:** Partial depletion of rods 3 dpl after focused and diffuse light lesions. Examples of remaining rods are indicated by arrows. **I, J:** Regenerated UV cones in the ONL at 28 days after focused and diffuse light lesions. **K, L:** GFP+ rods regenerate in the appropriate layer after focused and diffuse light lesion. Scale bars represent 20 µm.

Photoreceptor regeneration was assessed in retinal sections of the transgenic reporter fish opn1sw1:GFP and rh1:GFP respectively. The regular mosaic pattern of GFP+ photoreceptors in unlesioned retinae varied slightly in cell density depending on the retinal area (Fig. 10A–D). Data was acquired in the respective area of maximum lesion extent (anterior-central after focused light lesions and central-ventral after diffuse light lesions, see Fig. 3). At 3 dpl the GFP+ UV cones were completely ablated in zone 1 (Fig. 10E, focused light) and zone 2 (Fig. 10F, diffuse light). In regenerated retinae at 28 dpl, UV cones were less regularly shaped and organized compared to the untreated control (Fig. 10 I, J, S3). This is consistent with observations from previously published low intensity light lesion paradigms that also showed an imperfect regeneration of cones with respect to their distribution in a mosaic pattern [10], [45]. To compare the density of GFP+ UV cones in untreated and regenerated flatmounted retinae, respectively, we used the watershed algorithm of Fiji software to separate adjacent cells in the images (Fig. S3E). No significant increase in the number of photoreceptors per area was found compared to untreated control eyes (Fig. S3C).

Loss of rod photoreceptors was not complete at 3 dpl (Fig. 10H, K, arrows). Instead, some GFP+ rods survived in the ONL after both focused and diffuse light lesion. Regenerated rods became apparent by GFP expression after 4 dpl and constituted a complete layer of photoreceptors in the ONL after 28 days of regeneration (Fig. 10I, L). However, as observed in cone regeneration, regenerated rod appearance slightly differed with respect to density and regularity of the cell patterning compared to control.

In summary, these data indicate that after light lesions, both the completely ablated UV cones and partly ablated rods are regenerated within 28 dpl. Their density and patterning is almost, though not fully restored.

### Other neuronal cell types are affected in light lesions

**Figure 11:**
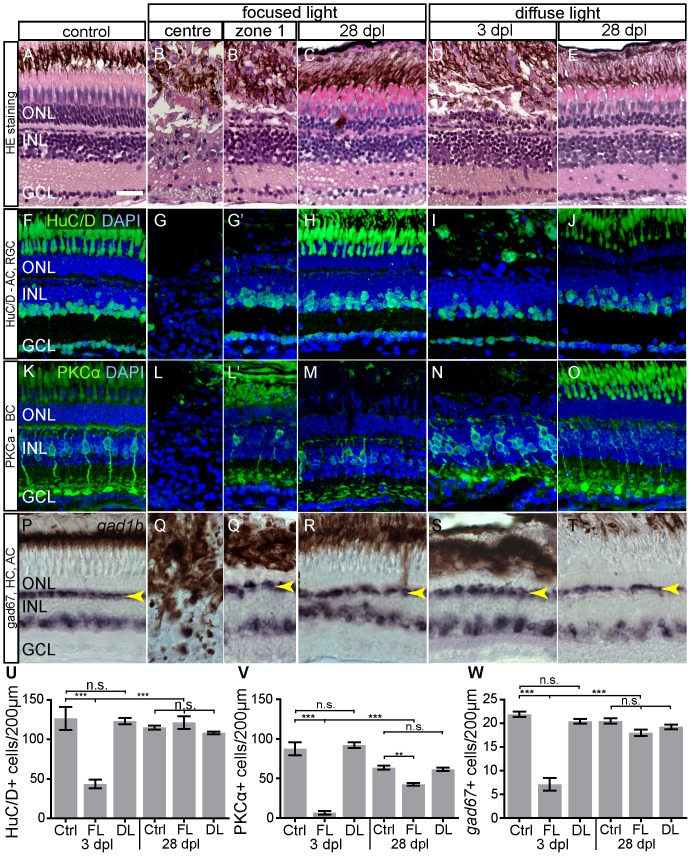
Morphology and Immunohistochemistry of neuronal cell types in light lesions at 3 and 28 dpl. **A–E**: Light lesioned retina morphology shown by hematoxylin/eosin staining. Severe damage to all laminae is found in the centre of focused light lesion zone 1 in an area of ∼50 µm in diameter. **F–J**: HuC/D antibody staining labels nuclei of AC and RGC. **K–O**: Antibody-staining against Phospho-Kinase C α-subunit (PKCα) labels a subset of bipolar cells. **P–T:** HC and a subset of AC are labelled by *gad1b in situ* hybridization. The continuous line pattern of HC is indicated by the arrowhead. **U–W:** Quantification of inner retinal neurons. The bars indicate the number of marker+ cells in 200 µm length of the retina across the most severe lesion. As control served untreated eyes of focused light treated fish. Regeneration of all cell types was assessed at 28 dpl. Error bars indicate SEM; ***p<0.001; AC: amacrine cells, BC: bipolar cells; DL: diffuse light lesions; FL: focused light lesions; GCL: ganglion cell layer; HC: horizontal cells; INL: inner nuclear layer; PR: photoreceptor cells; RGC: retinal ganglion cells. Scale bar represents 20 µm.

We found above that after focused light lesion, dying cells within the INL (Fig. 6 M). We confirmed this result with hematoxylin/eosin (HE) stainings, showing massive cell loss and misarrangement of the layers (Fig 10A, B, B′). These data indicate that not only photoreceptor cells might be affected by the high intensity UV light in the centre of zone 1 lesion. To test the survival of other cell types and determine the specificity of light lesions as tool for photoreceptor ablation, we quantified the expression of retinal cell markers in the two lesion paradigms and compared our results to histology data (Fig. 11A–E). We compared the number of marker-positive cells in unlesioned retinae to focused and diffuse light lesioned retinae at 3 dpl and after 28 days of regeneration.

To label amacrine cells (AC) and retinal ganglion cells (RGC) we used HuC/D as marker [46] (Fig. 11F). Three days after focused light lesions, a significant loss of cells was found in the center of the lesion (Fig. 11G), but not in adjacent zone 1 lesions (control: 126.6±14.68; center: 43.4±5.52; n = 3; p<0.0001; Fig. 11G′, U). After 28 days of regeneration, HuC/D+ cells are regenerated in focused light lesions (control: 115±2.38; center:121.3±7.86; n = 3; p<0.0001; Fig. 11H, U). We found no significant difference in HuC/D+ cell numbers in diffuse light lesions at either 3 dpl (Fig. 11I, U) or 28 dpl (Fig. 11J, U).

Other cell types of the neural retina such as horizontal cells (HC) and bipolar cells are spatially closer to the light lesion in the ONL than AC and RGC. Therefore, they might seem more likely to endure unspecific damage or die from secondary effects after a light lesion. We used Phospho-Kinase-C alpha (PKCα) antibodies to label selectively ON-bipolar cells with large axon terminals ramifying deep in the IPL [47], [48]. Control samples show the strictly perpendicular arrangement of PKCα+ cells in the INL (Fig. 11K). Only in the focused light lesion centre bipolar cells are lost at 3 dpl, whereas most of them survive in the adjacent light lesion zone 1. Although PKCα+ cells regenerated at 28 dpl, their number is smaller compared to untreated controls (Fig. 11M, V). In diffuse light lesions the pattern and numbers of PKCα+ cells are not affected (Fig. 11N, O, V).

The 67 kD isoform of the enzyme glutamic acid decarboxylase (Gad67) is encoded by *gad1b* and labels HC and AC in the adult zebrafish retina [49]. In situ hybridization against gad1b mRNA labels HC in a continuous linear pattern (Fig. 11P, arrowhead). As expected, the gad1b+ cells are lost in the centre of focused light lesions but survive in adjacent zone 1 (Fig. 11Q, Q′). Regenerated retinae still have irregularities in the *gad1b* expression pattern but quantification shows that the normal number of HC regenerated (Fig. 11R, W). Apart from some misarrangements of the gad1b+ linear pattern there was no significant loss of HC in diffuse light lesions (Fig. 11S, T, W)

Our results show that all assayed cell types persist in diffuse light lesions and in most areas of focused light lesions. The only loss of marker-positive cells occurs in the very centre of light lesions where light damages with highest intensity. All cell types regenerate within 28 days after the light lesion.

### In vivo imaging of degeneration and regeneration following diffuse and focused light lesion

Spectral domain optic coherence tomography (SD-OCT) is a new method of non-invasive *in vivo* imaging. It allows continuous observation of tissue morphology during degeneration and subsequent regeneration *in vivo* within the same animal. OCT uses long wavelength light that penetrates the surface of tissue with minimal reflections or scattering. The reflected light is recorded and converted to an image depicting the different optical properties (light scattering) of the tissue. Axons of the plexiform layers in the retina scatter light more than nuclear layers and therefore appear brighter in the image. Pigmented cells in the RPE and photoreceptor OS are also strongly light scattering and appear as bright signals in the OCT image. This method has been used before in medical diagnostics as well as in other animal models and was only recently adapted to zebrafish [50]. We have independently developed a custom-made OCT set-up and observe similar results as previously published. Furthermore, our system [32] was adapted to the optical properties of the fish eyes determined in the Zemax model (Fig. 4).

**Figure 12:**
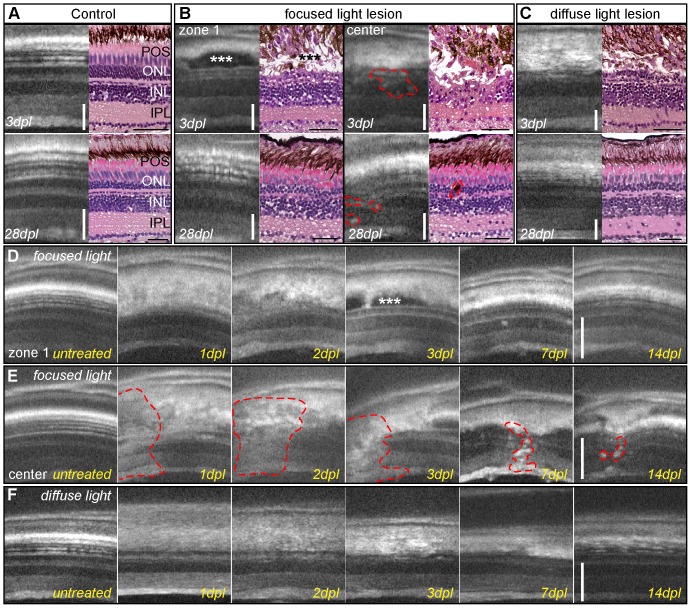
Imaging of light lesions with SD-OCT. **A**: Comparison of OCT images to HE staining, showing nuclear layers in dark shades and plexiform layers in grey. RPE cells appear as a thick white band over the thinner white spots of POS. **B:** Focused light lesions show strong heterogeneity, at least two different morphological areas are found in OCT and HE: zone 1 (left panel) with an intact inner nuclear layer, elongated nuclei (arrow) and accumulation of non-light-scattering material between ONL and RPE (asterisk). The center of focused light lesions (right panel) shows disorganization in all retinal layers between GCL and RPE (dashed line). Regenerated retina showing original thickness and morphology in zone 1 (left) but small inclusions in the center of focused light lesion (right) **C:** Homogeneous lesion at 3 days after diffuse light lesions in OCT and HE staining with a characteristic loss of ONL. Regenerated retinas closely resemble the morphology of control samples. **D, E:** Time course of the two typical morphologies after focused light lesion: the accumulation of material in zone 1 (3 dpl, asterisk) was cleared away later, whereas the center still shows small inclusions and flaws compared to the original structure (E, dashed line.) **F:** Time course of diffuse light lesions showing disorganization of POS and RPE at 1 and 2 dpl followed by a loss of the ONL at 3 dpl and regeneration of ONL and POS at 14 dpl. GCL: ganglion cell layer; HE: hematoxylin/eosin; INL: inner nuclear layer; IPL: inner plexiform layer; ONL: outer nuclear layer; POS: photoreceptor outer segments; RPE: retinal pigmented epithelium; SD-OCT: spectral domain optic coherence tomography. Scale bar represents 50 µm.

Using this OCT set-up, we directly compared the *in vivo* imaging to HE stainings. OCT imaging does not change the retinal structure over the time course of one month as shown in the untreated control (Fig. S4). Similar to previous results in light lesion experiments [4], [14], a reduction of nuclei in the ONL by 3 dpl compared to untreated control was found in OCT and morphological staining with HE (Fig. 12A, B, C). After a focused light lesion the layered structure of the retina was highly deranged. The RPE layer contained unstructured pigmented cell processes and cell debris. Loss of cells in all retinal layers in the center of focused light lesions indicates unspecific damage to non-light sensitive cell types, as described above (Fig. 12B, upper right panel and E). Compared to focused light lesions, the damage to ONL nuclei was less severe in diffuse light lesions and very homogeneous (Fig. 12C and F). The INL remained intact but elongated and displaced nuclei are found, similar to zone 1 and 2 in focused light lesions at 3 dpl (Fig. 12C, arrow). The images obtained from SD-OCT reflected the results from histological sections with respect to the layer structure, although single cell nuclei cannot be resolved. The repeated imaging of individual fish in a time course after both lesion paradigms however allows observation of a distinct lesion and the actual swelling or shrinking of the retina to occur in individual fish undergoing regeneration. In zone 1 of focused light lesions for instance, we found an accumulation of non-light scattering material between photoreceptor OS and ONL (Fig. 12B, D asterisk). The central spot of focused light lesions was characterized by strongly light scattering inclusions that partly remained throughout the whole time course of regeneration (Fig. 12E, and B lower right panel, dashed line). Diffuse light lesions showed a very homogeneous morphology throughout degeneration and regeneration in OCT images (Fig. 12F). At 7 dpl the retina also appeared thinnest compared to control without any photoreceptor OS and a very thin ONL. At 28 dpl not only the ONL and photoreceptor OS have regenerated, but also the retinal thickness has returned to normal (Fig. 12C lower panel). Thus, SD-OCT is a powerful method to image regeneration in the light lesioned adult retina.

## Discussion

Light lesions of photosensitive cells are a well-established tool to study regeneration of neurons in the adult vertebrate retina [4], [10], [17], [51]. In this study, we characterized and compared two different light lesion paradigms. The results showed an early onset of cell death, followed by a proliferation response of cells in the INL. Further, we showed unspecific damage of neurons only in focused light lesions, and only in the beam center. The advantages and disadvantages of both paradigms are discussed below.

UV radiation emitted from the fluorescent microscope lamp in both lesion paradigms quickly damages photosensitive cells, and to a lesser degree other neurons and the retinal pigmented epithelium (RPE). UV light is essential to induce light damage when a short exposure time is used. We applied selective wavelengths with similar light intensity but without using UV light, and could not detect a light lesion (data not shown).

In mammals, retinal light damage served to understand human retinal degeneration arising from environmental insult but also genetic diseases such as retinitis pigmentosa and age-related macular degeneration (reviewed by [52]). The molecular mechanism of light on disease progression was primarily addressed by applying photochemical stress via relatively low light intensities for several days to weeks. Light from within the absorption spectrum of the visual pigment leads to the so called class I damage mostly to photoreceptors. In contrast, class II damage is caused by brief exposures to short-wavelength light of high intensity which causes initially RPE damage (reviewed by [53]). With its brief exposure to short wavelength light, our focused light lesion model closely corresponds to a class II damage model. However, we did not notice extensive damage to RPE cells as reported in rabbit retinae [54]. Short exposure times are beneficial to study the defined onset and strict temporal series of events that follows a lesion using intense light, and therefore this paradigm is more suitable, for instance, to study changes in gene regulation. This would be more difficult in the low light lesion paradigm due to its slow and continuous progression. Additionally there is no need to use albino fish in intense light lesions. Both of our light lesion paradigms kill photoreceptors in pigmented fish, although strongly pigmented eyes showed milder light damage, similar to observations in rats [55], [56].

Intense light treatments on anaesthetized, immobilized fish were performed before [16], but without the use of a microscope and only for a very short exposure time. In contrast, the low light intensity paradigm uses a very long exposure time of one to seven consecutive days [10], and corresponds therefore rather to Class I damage. This has the advantage of less undesired effects on other cell types that are not light sensitive. However, a disadvantage of this method is continuous cell death and simultaneous overlapping regeneration.

### Light lesions induce damage in characteristic patterns

We measured the extent of damage in the light lesions using double *in situ* hybridization for all photoreceptor subtypes and made several unexpected observations.

Already in GFP transgenic fish, the different lesion patterns showed that GFP+ UV cones are more susceptible to light damage than GFP+ rods in both light lesion paradigms, which was confirmed by *in situ* hybridization on WT sections. In both lesion paradigms the extent of short wavelength sensitive cone ablation is higher than that of rods or red/green double cones. Also the strict counting of cell bodies, analogous to previously published data [18], yields 98% ablation of UV cones and only partial reduction of rods dorsal of the optic nerve head. At first glance, this result seems to contradict our previous data on lesion extents, where loss of rods was found mainly in the ventral retina (Table 4). However, since the dorsal counting area is just adjacent to the optic nerve (which locates within the ventral half of the retina in our sections), most of the rod lesion still locates ventral from the mathematical midline of the retina sections. A similarly peripheral dorsal counting area would have been outside the diffuse light lesion and yielded no loss in rods at all. Therefore, the result of the dorsal counting area should be understood as absolute cell numbers within the centre of the light lesion, rather than the result from a predefined, random dorsal area.

In contrast, the study of UV induced light lesions in albino fish [18] found only around 50% reduction of UV cones and 88% reduction of rods in the dorsal counting area. The lower number of ablated UV cones could be in part due to a slight difference in experimental conditions. We count loss of cells at 3 dpl when all cell debris is completely removed. However, counting at 2 hpl might impede discrimination of living and dead UV cones because of the major disorganisation in cone cell morphology.

The higher persistence of rods during intense light lesions might be due to light adaptation. During light adaptation, RPE granules migrate apically between rod OS and cone OS. This protects rod OS by scattering light back to cone OS [36]. At the same time rod OS elongate, while cone OS contract. In addition, melanin inside the pigment granules is excited by blue and UV light (reviewed by [57]), leading to accumulation of heat and photo-oxidative stress near the contracted cones. This effect is not happening in albino fish that lack melanin granules. Therefore the differential susceptibility of rod cells in response to diffuse light lesions seems to depend on pigmentation in the RPE. We found a gradual transition in rod density between partially depleted areas and areas with intact rod pattern, which is consistent with findings in albino fish [18]. In contrast, cones always show an abrupt transition between lesioned and non-lesioned areas; only in very rare cases a single cell survives within the lesion. This suggests that cone cell loss has a cooperative element to it.

A second unexpected result of our studies is that regional patterns become apparent after light lesion. Those are the consequence of the optical properties and anatomy of the fish eye, and its relative position to the light source. The optical properties of the sphere shaped lens in fish [29], [58] are especially adapted for vision in water and allow focusing parallel light beams with low spherical aberration. In focused light lesions, stationary light is always refracted to the same area in the central retina, leading to an accumulation of photo-oxidative and thermal stress in a central light lesion, especially in the focus. A gap between lens and iris allows some light to pass without refraction, which potentially increases lesion size in photoreceptors that are more prone to light damage. In focused light lesions, the fish eye is slightly tilted towards the anterior side so the focus of the lesion is shifted slightly to the anterior part of the retina. Measurement of fish eye dimensions and simulation of the fish eye as an optical system using Zemax software confirmed the location of the central lesion but could not explain the dorsal and anterior location of the peripheral light lesion. However, the light beam of the microscope is tilted in a 16° angle towards dorsal orientation when it meets the eye. Preliminary experiments suggest an increase of thermal stress in focused light lesions caused by out-of-pupil light that penetrates the eye through the surrounding skin and sclera in the dorsal rim of the retina. This probably contributes to formation of the peripheral semicircular lesion (data not shown). In diffuse light lesions, light reaches the retina from all directions, due to the swimming movement. The lens focuses the light in the central retina and therefore it is stronger affected by light damage compared to the peripheral retina. The horizontal orientation of the lesion stripe is caused by the swimming movement of fish and their orientation towards the light source. Similarly, the anterior vs. posterior extent of lesion might be influenced be the tendency of fish to swim away, parallel or towards the source of light.

### Retinal degeneration is precisely timed

Since the focused light lesion paradigm using UV light has short exposure times, it was possible to conduct temporal studies of the degeneration and regeneration processes with higher resolution and with comparison to non-lesioned control areas in the same retina/individual. We found that the short light exposure of 30′ led to a distinct time window in which cell death occurs, beginning at 8 hpl with a peak in cell death at 24 hpl. Focused light lesions seem to lead to an earlier initiation of cell death indicated by a second peak of TUNEL+ cells at 8 hpl. TUNEL staining shows mainly cell death of photoreceptors in the ONL as confirmed by loss of cells in morphological data. The only exception is the very central point of a focused light lesion that shows also significant cell death in the INL and GCL. Tissue damage in the INL and the GCL in the focused light lesion center are likely radiation side effects including accumulation of heat by excitation of melanin in RPE cells [59]. Secondary effects of photoreceptor cell death may however also contribute to apoptosis and necrosis in neurons, e.g. due to a missing contact dependent survival signal, or imbalances in homeostasis caused by cell debris. In conclusion, we found a clear difference between the two lesion paradigms: while diffuse light lesions lead to homogenous, photoreceptor specific lesions with steadily increasing cell death, there is local damage that affects cells in all retinal layers in focused light lesions leading to a faster onset of cell death. Therefore, focused light lesions have similar characteristics as other local injury models.

Notably, MG show an uptake of TUNEL+ cells at all timepoints and across the entire lesioned area in both lesion paradigms. The fact that no TUNEL staining is detected in the nucleus of the MG and that also photoreceptor derived GFP can be found in MG (data not shown) indicate that these cells do not die, but in contrast take up photoreceptor cell debris, as described before [39]. The number of phagocytic MG strictly correlates to the extent of lesioned area. Whereas a gradual spreading of TUNEL+ photoreceptors in the ONL results in increasing numbers of MG in diffuse light lesions, the locally confined focused light lesion fails to recruit more MG at later time points although more TUNEL+ cells were found in the same local lesions. In contrast, the number of L-Plastin+ leucocytes increased after both lesion paradigms up to 24 hpl corresponding to the peak of TUNEL+ cells indicating a stress dependent immune response during degeneration in the light lesioned retina.

### UV cone removal is not synchronous in diffuse light lesions

We found a striking difference in clearance of GFP+ photoreceptor debris between areas of strong and mild light damage. While short UV cones are already early removed in peripheral lesion areas, they remain as disrupted debris in central areas, where also other photoreceptors died. The clearance of photoreceptor debris correlates with the distribution of pigmeted processes of RPE cells. Under normal light conditions, rods and cones produce new photoreceptor OS to maintain a constant length (reviewed by [60]). RPE cells engulf shed photoreceptor OS and digest them. This effect is observed in both lesion paradigms, although it can be seen more clearly in diffuse light lesions where the 2 zones are larger. This observation suggests that pigment granule distribution in RPE processes can be actively attracted by a signal from dying cells and correlates with endocytosis or degradation of incorporated material within the RPE cell.

### Reactive proliferation after light damage

Since MG are thought to give rise to progenitor cells which regenerate all lost cells in the zebrafish retina, it was important to compare the kinetics of MG activation in the various light lesion paradigms. Generally, we find that both light lesion paradigms activate MG proliferation. As reported before, proliferation initiates between 2 and 3 dpl in the INL [51]. Also in both methods examined in this study, the response is in a defined time window with a maximum of proliferation at 3 dpl and almost none before 24 hpl and after 14 dpl. In zebrafish, MG respond to all different kinds of retinal injury with subsequent proliferation and thereby generate progenitor cells that are able to differentiate into all cell types of the neural retina [44]. These progenitors are visible as clusters of BrdU+ cells from 3 dpl to 7 dpl. More cells react to damage in the big, homogenous diffuse light lesion compared to focused light lesions. Although the absolute number of proliferating cells in focused light lesions is lower than in diffuse light lesions, the density of them within the lesion site is similar.

The morphology of MG in light lesions indicates that they do not only produce progenitors but also themselves react to damage. In the focused light lesion center, MG cells display swollen cell bodies and enlarged nuclei. Morphologically, these cells resemble gliotic MG in mammalian retinae (reviewed by [61]). Surprisingly only few MG were lost, although they are in contact with the calculated focus point of the eye model. The collapsed processes of MG in the ONL seem to delimit the neural retina from the subretinal space. The morphological changes of MG in light lesions at 3 dpl correlate with their activity in the proliferation response. The persistence of morphological alterations of MG indicates that they did not return to their normal state at 28 dpl.

### Loss and regeneration of inner retinal neurons

In focused light lesions we not only found loss of photoreceptors but also unspecific damage to neurons in the INL and GCL, so we investigated the loss and regeneration of inner retinal neurons in both paradigms. Thereby we were facing different challenges: On the one hand there are 5 different cell types with numerous subtypes that cannot all be labelled and counted separately. On the other hand it is also not possible to follow individual cells during the time course to see if they persist and get replaced at any time. Amacrine cells, ON-bipolar cells and horizontal cells were quantified within a fixed lesion area. Most non-light sensitive neuronal cell types in the retina were not severely affected after diffuse light lesions, neither at 3 dpl nor after 28 days of regeneration.

In contrast, morphology and cell death data of focused light lesions showed that the laminar structure and most cells were lost in the center area at 3 dpl. Therefore it is not surprising to find a decreased number of marker+ cells there at 3 dpl. The center area is filled with extracellular material, surviving MG cells and few, randomly arranged nuclei are found.

All respective cell types regenerate in focused light lesions. In conclusion we found – similar to photoreceptors – a regenerated retina that is similar to untreated controls.

### In vivo imaging in light lesioned fish

Optical coherence tomography is well established in clinical application, especially for the diagnosis, staging and monitoring of ocular diseases (reviewed by [62]). Recently the technology was adapted to small rodents as a non-invasive measurement tool for retinal degeneration models [63]. The good correlation of OCT data to histology was confirmed in mouse [64] and rat [65]. An important finding of our work, similar to what was reported by [50], is that SD-OCT can also be used as a powerful *in vivo* imaging tool to monitor retinal regeneration in fish, using our customized system. In human retinal imaging, the collimated sample beam is conventionally relay-imaged onto the pupil by means of a telescope lens configuration and focused onto the fundus by the eye itself. However, it is reasoned by others that this method is not optimum for small animals such as mice, because the small size and short focal length of the eye increases optical aberration, which degrades the OCT signal in terms of sensitivity and lateral resolution and thus, reduces image quality. Alternatively, a contact lens made from a microscope coverslip and a contact glass liquid is used, which effectively removes the refractive power of the air-corneal interface and thus, eliminates corneal aberration [66], [67]. Due to this procedure, the eye is made hyperopic. This facilitates the use of a convergent sample beam that can be easily focused on to the retina by simply adjusting the working distance between the scanning unit and the eye. These findings can also be transferred to the zebrafish model. For convenient imaging, the eye should be slightly hyperopic, which seems to be the case for anaesthetized fish in our experiments.

SD-OCT images obtained from regenerating fish retinae closely recapitulate the results of histology in terms of affected cell layers and lesion homogeneity. Additionally, we found an accumulation of as yet unidentified material in the histological stainings as well as in our OCT images. This material is located in the gap between RPE cells and photoreceptors, indicating that this is probably not an artifact of histological staining. In the future, monitoring light lesions using OCT imaging will be very useful because it allows non-invasive screening of morphological characteristics.

### Comparing the two light lesion paradigms

Focused light lesions offer, as a major advantage, a locally confined lesion next to unlesioned control areas within the same retina, in addition to an untreated control eye. Focused light lesions also cause, in their center, damage of RPE and INL, which can be used to asses regenerative properties of non-photoreceptor cell types of the retina. Unspecific damage to other cell layers can be reduced by lower light intensities or shorter exposure times. However, outside of the beam center, focused light lesion is photoreceptor specific without damage to other cell layers. The fact that only one fish can be light lesioned at a time and thus conditions of each experiment may differ slightly are disadvantages of focused light lesion.

Main characteristics of the diffuse light lesion are the large area of cone depletion and the greater homogeneity as compared to the focused light lesion. Other cell layers are essentially unaffected, and up to 4 fish can be treated simultaneously. Also, in diffuse light lesions longer exposure times could be used, and would lead to more light damage, whereas the fish in focused light lesion experiments are anaesthetized for not more than 30 minutes. On the other hand, diffuse light lesions do not offer a proper control region of the retina within the same individual. Thus, both light lesion paradigms show advantages and disadvantages for studying regeneration in the vertebrate retina, the best choice depending on the precise experimental question.

## Supporting Information

Figure S1 **L-Plastin+ cells are enriched in light lesions at 24 hpl**. **A:** L-Plastin+ cells were found in various retinal cell layers after focused light lesions. **A′:** Double labeling with TUNEL (red) shows only minimum overlap and mostly complementary location of L-Plastin+ cells (cyan). **B, B′:** Diffuse light lesion analogous to A, A′. **C, D:** Time course of absolute number of L-Plastin+ cells found in focused (C) and diffuse light lesion (D) showing a peak at 24 hpl. Error bars indicate standard error of the mean; *** for p-values <0.001; ** for p-values <0.01; * for p<0.05.
(TIF)

Figure S2 **Quantification of Müller glia in light lesions.** The bars indicate the number of marker+ cells in 200 µm length of the retina across the most severe lesion. As control served untreated eyes of focused light treated fish. Regeneration was assessed at 28 dpl. Error bars indicate SEM; *p<0.05;**p<0.01.
(TIF)

Figure S3 **Changes in UV cone mosaic after regeneration in light lesions.** **A:** Close-up image of untreated control retina flatmount from opn1sw1:GFP fish. **B:** Close up image of regenerated retina (28 dpl) from the same line. **C:** Quantification of UV cones per area compared (n = 6; p = 0.28; error bars indicate SEM). **D, E:** Same as A, B after image modification in order to count the number of cones automatically with Fiji software. Scale bar represents 20 µm. **F, G:** *In vivo* image of UV cones from OCT Data before (F) and at 28 dpl after light lesion (G) from the same fish.
(TIF)

Figure S4 **Live imaging of an untreated control fish over the course of 29 days.** **A:** Histological staining of an untreated retina shows typical retinal layer structure. **B–H:** OCT images of the same fish acquired over 1 month shows no change in retinal structures. Scale bar represents 20 µm.
(TIF)

Table S1 **Primary Antibodies used for immunohistochemistry on retina sections.**
(DOCX)

Table S2 **Primers for amplification of in situ hybridization probes.**
(DOCX)

## References

[1] Brockerhoff SE, Fadool JM (2010) Genetics of photoreceptor degeneration and regeneration in zebrafish. Cell Mol Life Sci. 10.1007/s00018-010-0563-8 PMC3029675 20972813

[2] Otteson DC, Hitchcock PF (2003) Stem cells in the teleost retina: Persistent neurogenesis and injury-induced regeneration. Vision Res 43: 927. 12668062 10.1016/s0042-6989(02)00400-5

[3] Mensinger AF, Powers MK (1999) Visual function in regenerating teleost retina following cytotoxic lesioning. Vis Neurosci 16: 241. 10367959 10.1017/s0952523899162059

[4] Bernardos RL, Barthel LK, Meyers JR, Raymond PA (2007) Late-stage neuronal progenitors in the retina are radial muller glia that function as retinal stem cells. J Neurosci 27: 7028. 17596452 10.1523/JNEUROSCI.1624-07.2007 PMC6672216

[5] Fimbel SM, Montgomery JE, Burket CT, Hyde DR (2007) Regeneration of inner retinal neurons after intravitreal injection of ouabain in zebrafish. J Neurosci 27: 1712. 17301179 10.1523/JNEUROSCI.5317-06.2007 PMC6673754

[6] Fausett BV, Goldman D (2006) A role for alpha1 tubulin-expressing muller glia in regeneration of the injured zebrafish retina. J Neurosci 26: 6303. 16763038 10.1523/JNEUROSCI.0332-06.2006 PMC6675181

[7] Hochmann S, Kaslin J, Hans S, Weber A, Machate A, et al (2012) Fgf signaling is required for photoreceptor maintenance in the adult zebrafish retina. PLoS ONE 7: e30365. 22291943 10.1371/journal.pone.0030365 PMC3266925

[8] Cameron DA (2000) Cellular proliferation and neurogenesis in the injured retina of adult zebrafish. Vis Neurosci 17: 789. 11153658 10.1017/s0952523800175121

[9] Montgomery JE, Parsons MJ, Hyde DR (2010) A novel model of retinal ablation demonstrates that the extent of rod cell death regulates the origin of the regenerated zebrafish rod photoreceptors. J Comp Neurol 518: 800–814. 20058308 10.1002/cne.22243 PMC3656417

[10] Vihtelic TS, Hyde DR (2000) Light-induced rod and cone cell death and regeneration in the adult albino zebrafish (danio rerio) retina. J Neurobiol 44: 289. 10942883 10.1002/1097-4695(20000905)44:3<289::aid-neu1>3.0.co;2-h

[11] Shahinfar S, Edward DP, Tso MO (1991) A pathologic study of photoreceptor cell death in retinal photic injury. Curr Eye Res 10: 47. 10.3109/02713689109007610 2029848

[12] Li S, Chang CJ, Abler AS, Fu J, Tso MO, et al (1996) A comparison of continuous versus intermittent light exposure on apoptosis. Curr Eye Res 15: 914. 8921211 10.3109/02713689609017635

[13] Wenzel A, Grimm C, Samardzija M, Reme CE (2005) Molecular mechanisms of light-induced photoreceptor apoptosis and neuroprotection for retinal degeneration. Prog Retin Eye Res 24: 275. 15610977 10.1016/j.preteyeres.2004.08.002

[14] Vihtelic TS, Soverly JE, Kassen SC, Hyde DR (2006) Retinal regional differences in photoreceptor cell death and regeneration in light-lesioned albino zebrafish. Exp Eye Res 82: 558. 16199033 10.1016/j.exer.2005.08.015

[15] Bernardos RL, Raymond PA (2006) Gfap transgenic zebrafish. Gene Expr Patterns 6: 1007. 16765104 10.1016/j.modgep.2006.04.006

[16] Eichenbaum JW, Cinaroglu A, Eichenbaum KD, Sadler KC (2009) A zebrafish retinal graded photochemical stress model. J Pharmacol Toxicol Methods 59: 121. 19269339 10.1016/j.vascn.2009.02.006 PMC3884834

[17] Craig SE, Calinescu AA, Hitchcock PF (2008) Identification of the molecular signatures integral to regenerating photoreceptors in the retina of the zebra fish. J Ocul Biol Dis Infor 1: 73. 20072637 10.1007/s12177-008-9011-5 PMC2802516

[18] Thomas JL, Nelson CM, Luo X, Hyde DR, Thummel R (2012) Characterization of multiple light damage paradigms reveals regional differences in photoreceptor loss. Experimental eye research 97: 105–116. 22425727 10.1016/j.exer.2012.02.004 PMC3329775

[19] Ramachandran R, Reifler A, Parent JM, Goldman D (2010) Conditional gene expression and lineage tracing of tuba1a expressing cells during zebrafish development and retina regeneration. J Comp Neurol 518: 4196. 20878783 10.1002/cne.22448 PMC2948409

[20] Brand MG, Michael; Nüsslein-Volhard, Christiane (2002) Keeping and raising zebrafish. In: C.Nüsslein-Volhard, Dahm R, editors. Zebrafish, a practical approach. Oxford: Oxford University Press. pp. 7–37.

[21] Streisinger G, Walker C, Dower N, Knauber D, Singer F (1981) Production of clones of homozygous diploid zebra fish (brachydanio rerio). Nature 291: 293. 7248006 10.1038/291293a0

[22] Hamaoka T, Takechi M, Chinen A, Nishiwaki Y, Kawamura S (2002) Visualization of rod photoreceptor development using gfp-transgenic zebrafish. Genesis 34: 215. 12395387 10.1002/gene.10155

[23] Takechi M, Hamaoka T, Kawamura S (2003) Fluorescence visualization of ultraviolet-sensitive cone photoreceptor development in living zebrafish. FEBS Lett 553: 90. 14550552 10.1016/s0014-5793(03)00977-3

[24] Grandel H, Kaslin J, Ganz J, Wenzel I, Brand M (2006) Neural stem cells and neurogenesis in the adult zebrafish brain: Origin, proliferation dynamics, migration and cell fate. Dev Biol 295: 263. 16682018 10.1016/j.ydbio.2006.03.040

[25] Kroehne V, Freudenreich D, Hans S, Kaslin J, Brand M (2011) Regeneration of the adult zebrafish brain from neurogenic radial glia-type progenitors. Development 138: 4831. 22007133 10.1242/dev.072587

[26] Vihtelic TS, Doro CJ, Hyde DR (1999) Cloning and characterization of six zebrafish photoreceptor opsin cdnas and immunolocalization of their corresponding proteins. Vis Neurosci 16: 571–585. 10349976 10.1017/s0952523899163168

[27] Reifers F, Bohli H, Walsh EC, Crossley PH, Stainier DY, et al (1998) Fgf8 is mutated in zebrafish acerebellar (ace) mutants and is required for maintenance of midbrain-hindbrain boundary development and somitogenesis. Development 125: 2381–2395. 9609821 10.1242/dev.125.13.2381

[28] Schindelin J, Arganda-Carreras I, Frise E, Kaynig V, Longair M, et al (2012) Fiji: An open-source platform for biological-image analysis. Nat Meth 9: 676–682. 10.1038/nmeth.2019 PMC3855844 22743772

[29] Jagger W (1992) The optics of the spherical fish lens. Vision Res 32: : 1271<last_page> 1284. 10.1016/0042-6989(92)90222-5 1455702

[30] Verma Y, Rao KD, Suresh MK, Patel HS, Gupta PK (2007) Measurement of gradient refractive index profile of crystalline lens of fisheye in vivo using optical coherence tomography. Appl Phys B 87: 607–610.

[31] Kröger RHH, Campbell MCW (1996) Dispersion and longitudinal chromatic aberration of the crystalline lens of the african cichlid fish haplochromis burtoni. J Opt Soc Am A 13: 2341–2347.

[32] Cimalla P, Walther J, Mehner M, Cuevas M, Koch E (2009) Simultaneous dual-band optical coherence tomography in the spectral domain for high resolution in vivo imaging. Opt Express 17: 19486–19500. 19997169 10.1364/OE.17.019486

[33] Wolf-Schnurrbusch UE, Ceklic L, Brinkmann CK, Iliev ME, Frey M, et al (2009) Macular thickness measurements in healthy eyes using six different optical coherence tomography instruments. Invest Ophthalmol Vis Sci 50: 3432–3437. 19234346 10.1167/iovs.08-2970

[34] Cimalla P, Burkhardt A, Walther J, Hoefer A, Wittig D, et al.. Non-invasive imaging and monitoring of rodent retina using simultaneous dual-band optical coherence tomography. In: Fujimoto JG, Izatt JA, Tuchin VV, editors; 2011; San Francisco, California, USA. SPIE. pp. 788909–788907.

[35] Shaw SL, Yeh E, Bloom K, Salmon ED (1997) Imaging green fluorescent protein fusion proteins in saccharomyces cerevisiae. Curr Biol 7: 701–704. 9285714 10.1016/s0960-9822(06)00299-5

[36] Hodel C, Neuhauss SCF, Biehlmaier O (2006) Time course and development of light adaptation processes in the outer zebrafish retina. Anat Rec A Discov Mol Cell Evol Biol 288A: : 653<last_page> 662. 10.1002/ar.a.20329 16721865

[37] Bailey TJ, Fossum SL, Fimbel SM, Montgomery JE, Hyde DR (2010) The inhibitor of phagocytosis, o-phospho-l-serine, suppresses müller glia proliferation and cone cell regeneration in the light-damaged zebrafish retina. Experimental eye research 91: 601–612. 20696157 10.1016/j.exer.2010.07.017 PMC2962682

[38] Kassen SC, Ramanan V, Montgomery JE, T Burket C, Liu CG, et al (2007) Time course analysis of gene expression during light-induced photoreceptor cell death and regeneration in albino zebrafish. Dev Neurobiol 67: 1009. 17565703 10.1002/dneu.20362

[39] Bailey TJ, Fossum SL, Fimbel SM, Montgomery JE, Hyde DR (2010) The inhibitor of phagocytosis, o-phospho-l-serine, suppresses m√°ller glia proliferation and cone cell regeneration in the light-damaged zebrafish retina. Exp Eye Res 91: 601. 20696157 10.1016/j.exer.2010.07.017 PMC2962682

[40] Joly S, Francke M, Ulbricht E, Beck S, Seeliger M, et al (2009) Cooperative phagocytes: Resident microglia and bone marrow immigrants remove dead photoreceptors in retinal lesions. Am J Pathol 174: 2310–2323. 19435787 10.2353/ajpath.2009.090023 PMC2684195

[41] Schrijvers DM, Meyer GRYD, Kockx MM, Herman AG, Martinet W (2007) Comparison of apoptosis detection markers combined with macrophage immunostaining to study phagocytosis of apoptotic cells in situ. Biomarker Insights 1: 193–200. 19690649 PMC2716774

[42] Nguyen-Legros J, Hicks D (2000) Renewal of photoreceptor outer segments and their phagocytosis by the retinal pigment epithelium. Int Rev Cytol 196: 245–313. 10730217 10.1016/s0074-7696(00)96006-6

[43] Anderson DH, Fisher SK, Steinberg RH (1978) Mammalian cones: Disc shedding, phagocytosis, and renewal. Invest Ophthalmol Vis Sci 17: 117–133. 415019

[44] Raymond PA, Barthel LK, Bernardos RL, Perkowski JJ (2006) Molecular characterization of retinal stem cells and their niches in adult zebrafish. BMC Dev Biol 6: 36. 16872490 10.1186/1471-213X-6-36 PMC1564002

[45] Qin Z, Barthel LK, Raymond PA (2009) Genetic evidence for shared mechanisms of epimorphic regeneration in zebrafish. Proc Natl Acad Sci U S A 106: 9310. 19474300 10.1073/pnas.0811186106 PMC2695073

[46] Marusich MF, Furneaux HM, Henion PD, Weston JA (1994) Hu neuronal proteins are expressed in proliferating neurogenic cells. J Neurobiol 25: 143–155. 7517436 10.1002/neu.480250206

[47] Connaughton VP, Graham D, Nelson R (2004) Identification and morphological classification of horizontal, bipolar, and amacrine cells within the zebrafish retina. J Comp Neurol 477: 371. 15329887 10.1002/cne.20261

[48] Yazulla S, Studholme KM (2001) Neurochemical anatomy of the zebrafish retina as determined by immunocytochemistry. J Neurocytol 30: 551. 12118162 10.1023/a:1016512617484

[49] Connaughton VP, Behar TN, Liu WL, Massey SC (1999) Immunocytochemical localization of excitatory and inhibitory neurotransmitters in the zebrafish retina. Vis Neurosci 16: 483–490. 10349969 10.1017/s0952523899163090

[50] Bailey TJ, Davis DH, Vance JE, Hyde DR (2012) Spectral-domain optical coherence tomography as a noninvasive method to assess damaged and regenerating adult zebrafish retinas. Invest Ophthalmol Vis Sci 53: 3126–3138. 22499984 10.1167/iovs.11-8895 PMC3383185

[51] Thummel R, Kassen SC, Enright JM, Nelson CM, Montgomery JE, et al (2008) Characterization of müller glia and neuronal progenitors during adult zebrafish retinal regeneration. Exp Eye Res 87: 433. 18718467 10.1016/j.exer.2008.07.009 PMC2586672

[52] Organisciak DT, Vaughan DK (2010) Retinal light damage: Mechanisms and protection. Progress in retinal and eye research 29: 113. 19951742 10.1016/j.preteyeres.2009.11.004 PMC2831109

[53] Organisciak DT, Winkler BS (1994) Retinal light damage: Practical and theoretical considerations. Prog Retin Eye Res 13: 1–29.

[54] Lawwill T, Crockett S, Currier G (1977) Retinal damage secondary to chronic light exposure, thresholds and mechanisms. Doc Ophthalmol 44: 379–402. 413705 10.1007/BF00230089

[55] Rapp LM, Williams TP (1980) The role of ocular pigmentation in protecting against retinal light damage. Vision Res 20: 1127–1131. 7269270 10.1016/0042-6989(80)90050-4

[56] Noell WK, Walker VS, Kang BS, Berman S (1966) Retinal damage by light in rats. Investigative ophthalmology 5: 450. 5929286

[57] Glickman RD (2002) Phototoxicity to the retina: Mechanisms of damage. Int J Toxicol 21: 473–490. 12537644 10.1080/10915810290169909

[58] Bantseev V, Moran KL, Dixon DC, Trevithick JR, Sivak JC (2004) Optical properties, mitochondria, and sutures of lenses of fishes: A comparative study of nine species. Can J Zool 82: 86.

[59] Parver LM, Auker C, Carpenter DO (1980) Choroidal blood flow as a heat dissipating mechanism in the macula. Am J Ophthalmol 89: 641–646. 6769334 10.1016/0002-9394(80)90280-9

[60] Kevany BM, Palczewski K (2010) Phagocytosis of retinal rod and cone photoreceptors. Physiology 25: 8. 20134024 10.1152/physiol.00038.2009 PMC2839896

[61] Bringmann A, Pannicke T, Grosche J, Francke M, Wiedemann P, et al (2006) Muller cells in the healthy and diseased retina. Progress in retinal and eye research 25: 397. 16839797 10.1016/j.preteyeres.2006.05.003

[62] Walther J, Gaertner M, Cimalla P, Burkhardt A, Kirsten L, et al (2011) Optical coherence tomography in biomedical research. Anal Bioanal Chem 400: 2721–2743. 21562739 10.1007/s00216-011-5052-x

[63] Horio N, Kachi S, Hori K, Okamoto Y, Yamamoto E, et al (2001) Progressive change of optical coherence tomography scans in retinal degeneration slow mice. Arch Ophthalmol 119: 1329–1332. 11545639 10.1001/archopht.119.9.1329

[64] Huber G, Beck SC, Grimm C, Sahaboglu-Tekgoz A, Paquet-Durand F, et al (2009) Spectral domain optical coherence tomography in mouse models of retinal degeneration. Invest Ophthalmol Vis Sci 50: 5888–5895. 19661229 10.1167/iovs.09-3724 PMC2800101

[65] Nagata A, Higashide T, Ohkubo S, Takeda H, Sugiyama K (2009) In vivo quantitative evaluation of the rat retinal nerve fiber layer with optical coherence tomography. Invest Ophthalmol Vis Sci 50: 2809–2815. 19182247 10.1167/iovs.08-2764

[66] Kim KH, Puoris'haag M, Maguluri GN, Umino Y, Cusato K, et al. (2008) Monitoring mouse retinal degeneration with high-resolution spectral-domain optical coherence tomography. J Vis 8: : 17 11–11. 10.1167/8.1.17 18318620

[67] Srinivasan VJ, Ko TH, Wojtkowski M, Carvalho M, Clermont A, et al (2006) Noninvasive volumetric imaging and morphometry of the rodent retina with high-speed, ultrahigh-resolution optical coherence tomography. Invest Ophthalmol Vis Sci 47: 5522–5528. 17122144 10.1167/iovs.06-0195 PMC1941766

