# Supplementary figures and images for: Characterization of Light Lesion Paradigms and Optical Coherence Tomography as Tools to Study Adult Retina Regeneration in Zebrafish
Source: PLoS One. 2013 Nov 26;8(11):e80483. doi: 10.1371/journal.pone.0080483 (PMC3841302; doi:10.1371/journal.pone.0080483)

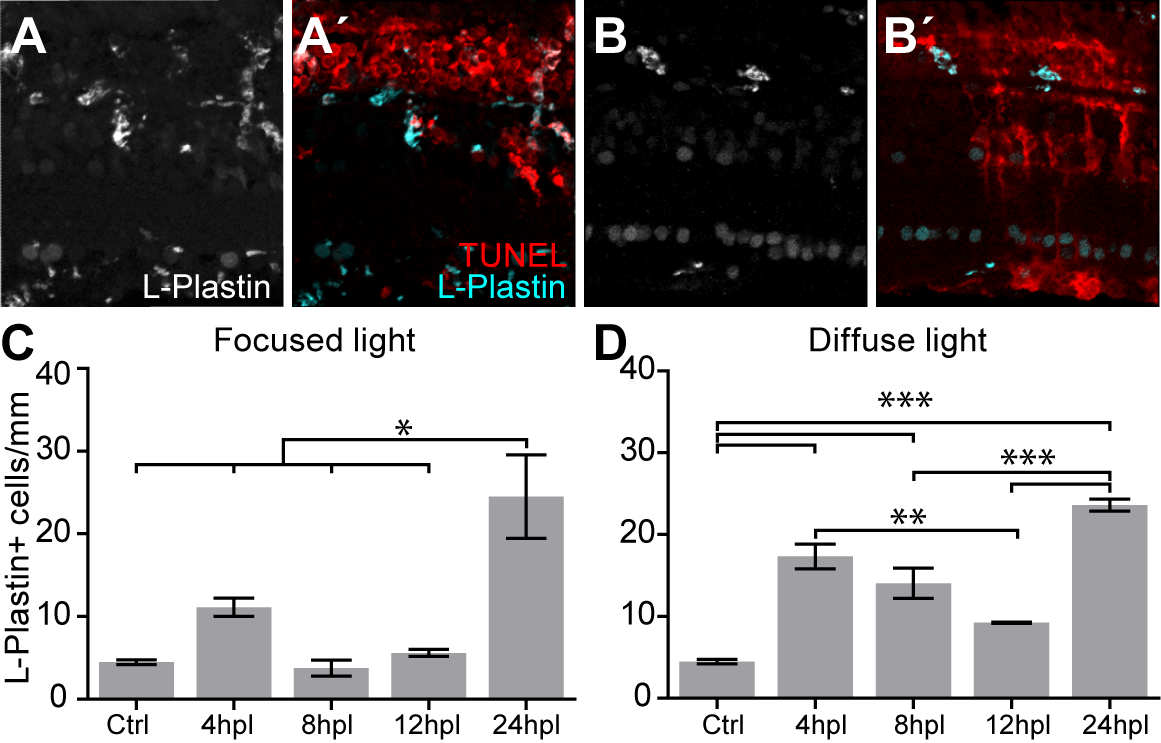

Supplement: Figure S1 — L-Plastin+ cells are enriched in light lesions at 24 hpl. A: L-Plastin+ cells were found in various retinal cell layers after focused light lesions. A′: Double labeling with TUNEL (red) shows only minimum overlap and mostly complementary location of L-Plastin+ cells (cyan). B, B′: Diffuse light lesion analogous to A, A′. C, D: Time course of absolute number of L-Plastin+ cells found in focused (C) and diffuse light lesion (D) showing a peak at 24 hpl. Error bars indicate standard error of the mean; *** for p-values <0.001; ** for p-values <0.01; * for p<0.05. (TIF) [file pone.0080483.s001.tif]

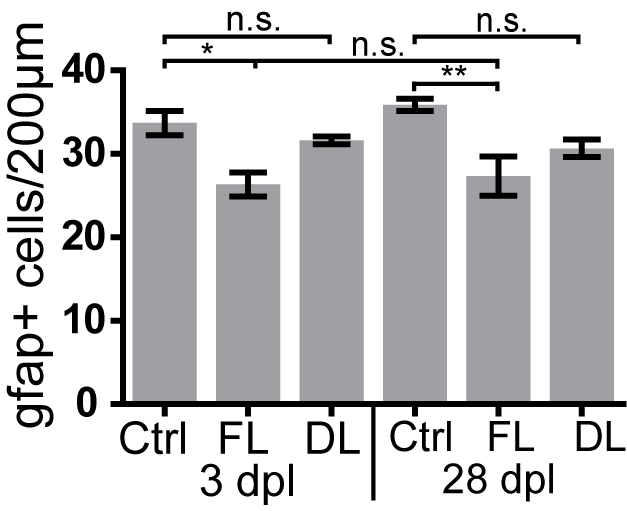

Supplement: Figure S2 — Quantification of Müller glia in light lesions. The bars indicate the number of marker+ cells in 200 µm length of the retina across the most severe lesion. As control served untreated eyes of focused light treated fish. Regeneration was assessed at 28 dpl. Error bars indicate SEM; *p<0.05;**p<0.01. (TIF) [file pone.0080483.s002.tif]

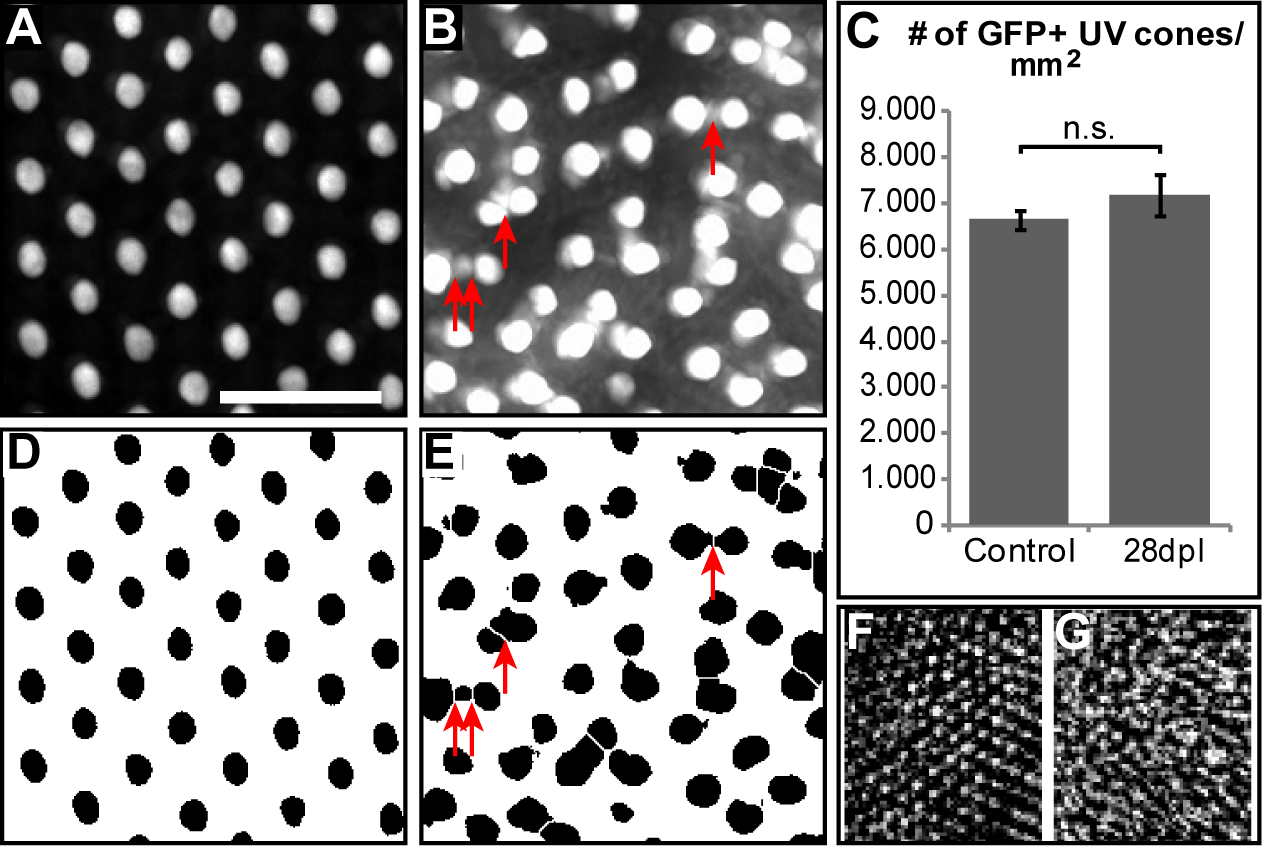

Supplement: Figure S3 — Changes in UV cone mosaic after regeneration in light lesions. A: Close-up image of untreated control retina flatmount from opn1sw1:GFP fish. B: Close up image of regenerated retina (28 dpl) from the same line. C: Quantification of UV cones per area compared (n = 6; p = 0.28; error bars indicate SEM). D, E: Same as A, B after image modification in order to count the number of cones automatically with Fiji software. Scale bar represents 20 µm. F, G: In vivo image of UV cones from OCT Data before (F) and at 28 dpl after light lesion (G) from the same fish. (TIF) [file pone.0080483.s003.tif]

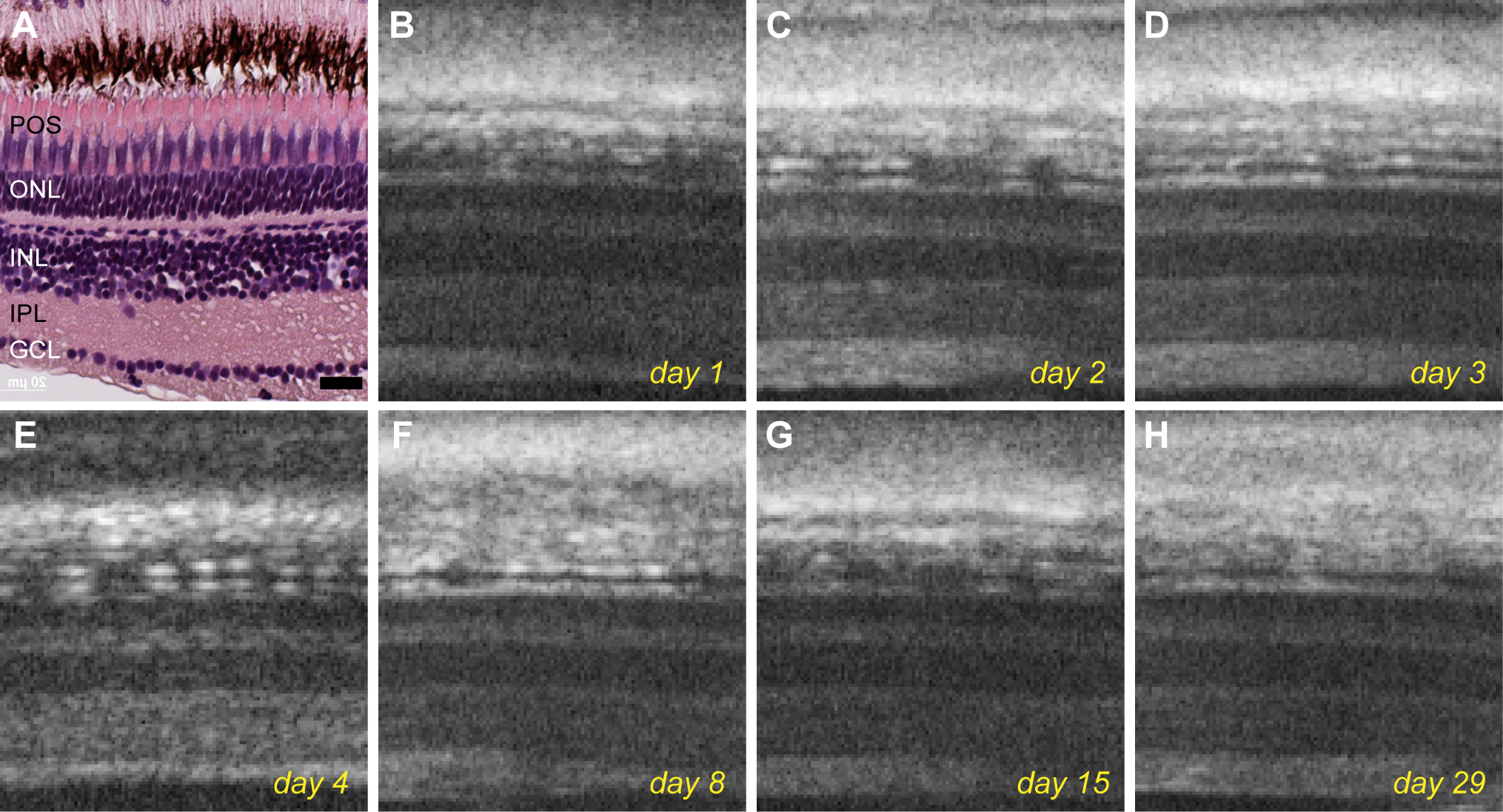

Supplement: Figure S4 — Live imaging of an untreated control fish over the course of 29 days. A: Histological staining of an untreated retina shows typical retinal layer structure. B–H: OCT images of the same fish acquired over 1 month shows no change in retinal structures. Scale bar represents 20 µm. (TIF) [file pone.0080483.s004.tif]
