# Supplementary material for: Characterization of Light Lesion Paradigms and Optical Coherence Tomography as Tools to Study Adult Retina Regeneration in Zebrafish
Source: PLoS One. 2013 Nov 26;8(11):e80483. doi: 10.1371/journal.pone.0080483 (PMC3841302; doi:10.1371/journal.pone.0080483)
Supplement: Table S1 — Primary Antibodies used for immunohistochemistry on retina sections. (DOCX) [file pone.0080483.s005.docx]

| **Antigen** | **Host** | **Company** | **Dilution** |
| --- | --- | --- | --- |
| BrdU | mouse | BD | 1:500 |
| BrdU | rat | Serotec | 1:1000 |
| GFP | rabbit | Molecular probes | 1:500 |
| Glutamine Synthetase | mouse | Millipore | 1:1000 |
| HuC/D | mouse | Molecular probes | 1:300 |
| L-Plastin | Rabbit | Shinomiya et. al., 2003[[1](#_ENREF_1)] | 1:8000 |
| PKCα | rabbit | Santa Cruz Biotechnology Inc. | 1:500 |
| Zpr-1 | mouse | ZIRC | 1:500 |
| Zpr-3 | mouse | ZIRC | 1:500 |

1. Shinomiya H, Nagai K, Hirata H, Kobayashi N, Hasegawa H, et al. (2003) Preparation and characterization of recombinant murine p65/l-plastin expressed in *escherichia coli* and high-titer antibodies against the protein. Bioscience, Biotechnology, and Biochemistry 67: 1368-1375.
