# Supplementary material for: Characterization of Light Lesion Paradigms and Optical Coherence Tomography as Tools to Study Adult Retina Regeneration in Zebrafish
Source: PLoS One. 2013 Nov 26;8(11):e80483. doi: 10.1371/journal.pone.0080483 (PMC3841302; doi:10.1371/journal.pone.0080483)
Supplement: Table S2 — Primers for amplification of in situ hybridization probes. (DOCX) [file pone.0080483.s006.docx]

| **Gene** | **Primers** | **Product Size** |
| --- | --- | --- |
| opn1lw1 | T3F: 5´ GAGGGTCCCAATTACCACATT | 282 bp |
|  | T3R: 5´TGACACAGTGTAGCCTTCAAAAA |  |
| opn1sw1 | T7F: 5´ GTTGACTCCACCAGGACACAC | 304 bp |
|  | T7R: 5´ TGACTTACAGAGAACGTGTCGAA |  |
| opn1sw2 | T7F: 5´ AGCCAAAGCTCAAGCTGATTC | 214 bp |
|  | T7R: 5´ GACGGGATTGTACACTGTAGAGG |  |
| rho | T7F: 5´ ATTCTACGTGCCTATGTCCAATG | 629 bp |
|  | T7R: 5´ CTATCAGTGGGATGAAGAAGTGC |  |
